# Mitochondrial DNA heteroplasmy is modulated during oocyte development propagating mutation transmission

**DOI:** 10.1126/sciadv.abi5657

**Published:** 2021-12-08

**Authors:** Haixin Zhang, Marco Esposito, Mikael G. Pezet, Juvid Aryaman, Wei Wei, Florian Klimm, Claudia Calabrese, Stephen P. Burr, Carolina H. Macabelli, Carlo Viscomi, Mitinori Saitou, Marcos R. Chiaratti, James B. Stewart, Nick Jones, Patrick F. Chinnery

**Affiliations:** 1 Department of Clinical Neurosciences, School of Clinical Medicine, University of Cambridge, Cambridge Biomedical Campus, Cambridge, UK.; 2 Medical Research Council Mitochondrial Biology Unit, University of Cambridge, Cambridge Biomedical Campus, Cambridge, UK.; 3 EPSRC Centre for the Mathematics of Precision Healthcare, Department of Mathematics, Imperial College, London, UK.; 4 Leverhulme Centre for Cellular Bionics, Imperial College, London, UK.; 5 Departamento de Genética e Evolução, Universidade Federal de São Carlos, São Carlos 13565-905, Brazil.; 6 Department of Anatomy and Cell Biology, Graduate School of Medicine, Kyoto University, Yoshida-Konoe-cho, Sakyo-ku, Kyoto 606-8501, Japan.; 7 JST, ERATO, Yoshida-Konoe-cho, Sakyo-ku, Kyoto 606-8501, Japan.; 8 Max Planck Institute for Biology of Ageing, Cologne 50931, Germany.; 9 Biosciences Institute, Faculty of Medical Sciences, Wellcome Centre for Mitochondrial Research, Newcastle University, Newcastle upon Tyne, UK.

## Abstract

Heteroplasmic mitochondrial DNA (mtDNA) mutations are a common cause of inherited disease, but a few recurrent mutations account for the vast majority of new families. The reasons for this are not known. We studied heteroplasmic mice transmitting m.5024C>T corresponding to a human pathogenic mutation. Analyzing 1167 mother-pup pairs, we show that m.5024C>T is preferentially transmitted from low to higher levels but does not reach homoplasmy. Single-cell analysis of the developing mouse oocytes showed the preferential increase in mutant over wild-type mtDNA in the absence of cell division. A similar inheritance pattern is seen in human pedigrees transmitting several pathogenic mtDNA mutations. In m.5024C>T mice, this can be explained by the preferential propagation of mtDNA during oocyte maturation, counterbalanced by purifying selection against high heteroplasmy levels. This could explain how a disadvantageous mutation in a carrier increases to levels that cause disease but fails to fixate, causing multigenerational heteroplasmic mtDNA disorders.

## Teaser

Single-cell analysis of mouse oocytes implicates the preferential transmission of a pathogenic mitochondrial DNA (mtDNA) mutation.

## INTRODUCTION

Over 100 different single-nucleotide variants of the 16.6-kilobase (kb) mitochondrial genome (mtDNA) cause mitochondrial disorders affecting ~1 in 8000 humans (*1*, *2*), but a very small number of independently recurring mutations present in the clinic, with m.3243A>G being overwhelmingly the most common, accounting for 80% of adults with a pathogenic heteroplasmic mtDNA mutation (*3*). The next most common heteroplasmic mutation in adults with mtDNA disease is m.8244A>G, found in only 3% of affected individuals (*3*). The reasons for this are not known. A reanalysis of 8391 high-depth human mtDNA sequences for variants present at >1% heteroplasmy (*4*) showed no enrichment for known pathogenic mutations among the low-level heteroplasmies (<5% heteroplasmy, *P* = 0.27; <2% heteroplasmy, *P* = 0.74). Thus, the prevalence of specific mtDNA mutations in clinics from the same geographic region (*3*) is not solely related to the frequency of de novo mutations in the population and, at least in part, is likely to reflect the propagation of preexisting low-level heteroplasmy to high-level heteroplasmy that causes disease.

Initially, all mtDNA mutations affect a proportion of the many mtDNA molecules within a cell (heteroplasmy). Heteroplasmic mtDNA variants segregate rapidly during maternal transmission because of a germline genetic bottleneck. In mice, humans, and other vertebrates, the fertilized single-cell zygote contains >100,000 mtDNA molecules per cell, falling to a few hundred molecules per cell in primordial germ cells (PGCs) forming the newly committed germ cell lineage (*5*–*7*). The reduction in cellular mtDNA content is sufficient to explain most of the variation in heteroplasmy seen in primary oocytes, which go on to form the next generation (*6*, *7*), but there is emerging evidence that other forces come in to play. The signature of selection has been seen in the offspring of several species (*8*–*11*), including humans (*4*), typically limiting the inheritance of deleterious mutations that compromise oxidative phosphorylation. This raises the question—If selection “purifies” the mtDNA population during germline development, how do pathogenic mtDNA mutations ever reach levels that cause human diseases?

Here, we show the signature of selfish mtDNA propagation in mice transmitting the m.5024C>T tRNA^Ala^ mutation on a haplotype with the m.13715C>T/ND6 variant (*12*, *13*). Analyzing single oocytes, we observed a preferential increase in mutant mtDNA during late oocyte development, where very high levels were also prevented by purifying selection. Although limited to one specific mtDNA mutation in mice that is rare in humans (*14*, *15*), the inheritance pattern resembles several different human pathogenic mutations, implying a that common mechanism exists across species and for more than one mutation. Our findings for the m.5024C>T tRNA^Ala^ mutation provide an explanation why and how a disadvantageous mtDNA haplotype can be preferentially transmitted to the next generation but fails to fixate in the female germ line, potentially leading to multigenerational heteroplasmic pedigrees.

## RESULTS

**Fig. 1.**
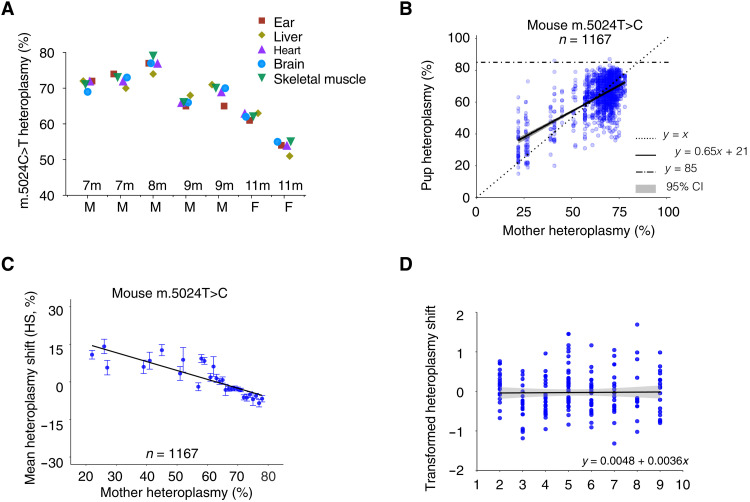
Heteroplasmy levels in *BVSC-*tRNA^Ala^ m.5024C>T mice. (**A**) Mitochondrial DNA heteroplasmy levels in the ear, liver, heart, brain, and skeletal muscle at 7, 8, 9, and 11 months (m) of age. M, male; F, female. (**B**) Transmission of heteroplasmy from 42 mothers to 1167 pups. *y = x* corresponds to the 1:1 transmission of heteroplasmy from mother to her offspring. *y = mx + c* corresponds to the line of best fit for the data with 95% confidence intervals for the regression line (gray). *y* = 85% corresponds to the threshold with no mice exceeding this heteroplasmy value. Error bars are SEM. (**C**) Difference in heteroplasmy levels (heteroplasmy shift) between the mothers and pups shown in (B). Mothers with low heteroplasmy levels tend to transmit higher levels to their offspring, and mothers with high heteroplasmy levels tend to transmit lower levels to their offspring. (**D**) Relationship between the maternal age at delivery and the transformed heteroplasmy shift in her offspring. *y* = *mx* + *c* corresponds to the line of best fit for the data with 95% confidence intervals for the regression line (gray, *P* = 0.5787), showing no change in the mean transformed heteroplasmy shift with maternal age (see Materials and Methods). Pups, *n* = 176; litters, *n* = 33; mothers, *n* = 15; where maternal ages (months) were available.

To explore potential mechanisms responsible for the preferential transmission of mutant mtDNA, we studied the m.5024C>T tRNA^Ala^ mutation in mice (*12*, *13*), which compromises oxidative phosphorylation when >90% of molecules are affected in cardiomyocytes and corresponds to the known m.5650G>A pathogenic mtDNA mutation in humans (*14*, *15*). First, we measured heteroplasmy levels in different organs at different ages (Fig. 1A), confirming that ear biopsy heteroplasmy levels are representative of mean tissue levels in different organs (*16*). Next, we measured heteroplasmy levels in 42 female mice and their 1167 offspring (Fig. 1, B and C). This showed evidence of positive selection when the mutation was transmitted from mothers with low heteroplasmy levels, and purifying selection against high heteroplasmy levels (>90%). A reconsideration of published data (*8*) from the m.3875delC tRNA^Met^ heteroplasmic mouse showed the same signature [figure 3 in (*8*)], which had not been previously noted, indicating that this pattern was not specific to one mtDNA mutation. In keeping with human data (*4*), the age of the mother did not influence the transmission of heteroplasmy (Fig. 1D).

### Single-cell mtDNA analysis shows positive and negative selection during oocyte development

**Fig. 2.**
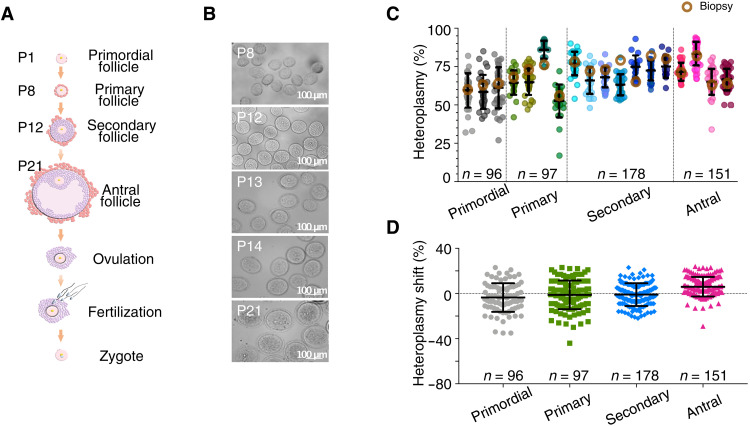
Heteroplasmy levels in developing single oocytes from *BVSC-*tRNA^Ala^ m.5024C>T mice. (**A**) Schematic representation of the major events during oocyte development and fertilization. (**B**) Representative image of manually picked P8, P12, P13, P14, and P21 oocytes. Scale bar, 100 μm. P, postnatal age/days. (**C**) m.5024C>T heteroplasmy levels in single oocytes isolated from the females. Mean ear biopsy, brown circle. P1 primordial follicle oocyte, *n* = 96. P8 primary follicle oocyte, *n* = 97. P12 secondary follicle oocyte, *n* = 178. P21 antral follicle oocyte, *n* = 151. All error bars are represented as means ± SD. (**D**) Difference in m.5024C>T heteroplasmy levels between single oocytes isolated from developing follicles and the corresponding maternal heteroplasmy level (heteroplasmy shift) showing the mean increase in antral follicles.

**Fig. 3.**
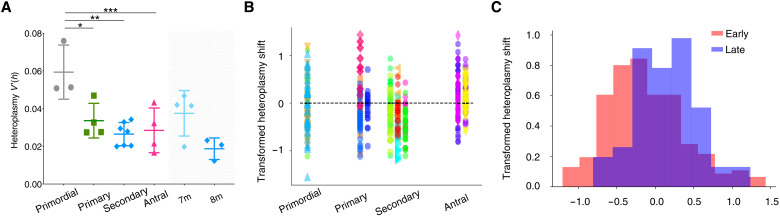
Evidence of positive selection during oocyte development in the *BVSC-*tRNA^Ala^ m.5024C>T mice. (**A**) Normalized variance of the m.5024C>T mutation at different stages of oocyte development: unshaded, neonatal mice; shaded, antral follicles collected from 7-month-old (*n* = 60) and 8-month-old (*n* = 54) females 7m. *V*′(*h*) = *V*_h_/μ(1 − μ). *V*_h_ is the heteroplasmy variance, and μ is the mean heteroplasmy. Each color corresponds to a different mouse. *P = 0.0172, **P = 0.001, and ***P = 0.0046. Error bars are represented as means ± SD. (**B**) Transformed m.5024C>T heteroplasmy shift during oocyte development = ln(*h*(*h*_0_ − 1)/*h*_0_(*h* − 1)). *h* is the oocyte heteroplasmy measurement, oocyte, or pup, and *h*_0_ is the corresponding mother’s ear biopsy. Each strip corresponds to a different day from left to right: P1, P8, P9, P12, P13, P14, P21, and P22. Each color corresponds to one of 18 different females. (**C**) Distribution of heteroplasmy shifts during early and late oocyte development. Early, primordial, primary, and secondary follicles; late, antral follicles.

Given that mammalian mtDNA is almost exclusively transmitted down the maternal line, we studied the female germ line to identify the underlying mechanisms. We crossed the m.5024C>T tRNAAla heteroplasmic mouse with Blimp1-mVenus and Stella-ECFP mice (BVSC, Stella = Dppa3) (*17*, *18*). The fluorescent transgene facilitated the isolation of single cells from each stage of oocyte development and maturation, particularly from the early stages (Fig. 2, A and B). We observed a range of heteroplasmy levels in 96 quiescent oocytes from primordial follicles, 275 growing oocytes from primary and secondary follicles, and 151 fully grown oocytes from antral follicles isolated from 18 mice (Fig. 2C and table S2). In oocytes from primordial follicles, the cell heteroplasmy levels fitted a Kimura distribution (*19*) (Fig. 2D, fig. S1, and tables S1 and S2), and the heteroplasmy variance (Fig. 3A) was similar to previous observations in mice transmitting polymorphic mtDNA variants (*20*), in keeping with a prenatal mtDNA genetic bottleneck. However, in growing (primary and secondary follicles) and fully grown oocytes (antral follicles), we saw an unexpected reduction in heteroplasmy variance (Fig. 3A), which was apparent in antral follicles isolated from neonatal (unshaded) and both 7- and 8-month-old (shaded) females. Normalizing the raw heteroplasmy values (Fig. 3B and fig. S2) to avoid bias due to the initial heteroplasmy value [h0; see Materials and Methods and (*21*)] suggested that negative selection in growing oocytes (primary and secondary follicles, *P* = 0.0034) was followed by positive selection in the antral follicles (*P* = 0.0014; Fig. 3C), but with no oocytes containing very high (>89%) heteroplasmy levels (Fig. 2C).

### Preferential replication of the mutant molecules and negative selection at high levels

**Fig. 4.**
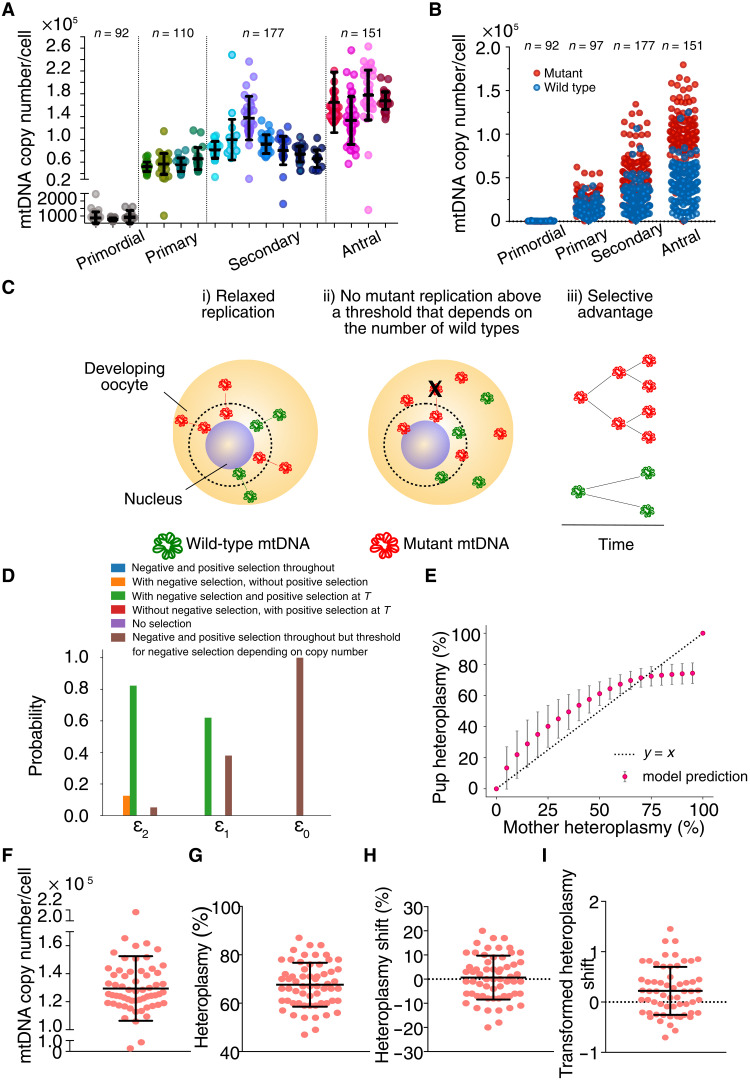
Single-cell mtDNA content during oocyte development in the *BVSC*-tRNA^Ala^ m.5024C>T mice. (**A**) mtDNA content in single growing oocytes. **(B)** Wild-type and m.5024C>T mutated mtDNA content in single growing oocytes. (**C**) Schematics of the favored model: fixed subset of replicating molecules R0, selective advantage for mutants λ*_m_* > λ*_w_*, and negative selection above a heteroplasmy threshold that depends on wild-type copy number (see Materials and Methods). (**D**) Statistical support for the models considered from ABC model selection (see Materials and Methods). As the threshold decreases from left to right, forcing a stricter agreement with experimental data, the support favors a model (green bars, *H* = 5) where there is a replicative advantage favoring mutant mtDNA and purifying selection acts against levels of mutation that depend on wild-type copy number (*H* = 0: with positive selection throughout, with negative selection; *H* = 1: without positive selection, with negative selection; *H* = 2: negative selection, with positive selection after *T*; *H* = 3: with positive selection after *T* and without negative selection; *H* = 4: without positive and negative selection). (**E**) Model prediction of the mean heteroplasmy in antral follicle oocytes after development for different initial heteroplasmy values. The error bars give the standard deviation generated by the top 200 accepted parametrizations. (**F** to **I)** Single-cell mtDNA analysis of ovulated oocytes from two 7-week-old *BVSC-*tRNA^Ala^ m.5024C>T mothers. (F) mtDNA heteroplasmy levels, (G) single oocyte heteroplasmy levels, (H) heteroplasmy shifts compared to the mothers’ ear biopsy, and (I) transformed heteroplasmy shifts compared to the mothers’ ear biopsy. *N* = 60. Bars show the mean ± SD.

To understand the molecular mechanism, we measured the amount of mtDNA in single cells during oocyte development. Mature mouse oocytes are known to contain ~10^5^ mtDNAs, but it is not known when most of the mtDNA replication occurs. We observed a marked increase in intracellular mtDNA content toward the late stages of oocyte development (primordial follicle mean = 347 molecules per cell, SD = 355.8; antral follicle mean = 1.41 × 10^5^ molecules per cell, SD = 4.38 × 10^4^; Fig. 3A), which appeared to favor mutant mtDNA at the lower heteroplasmies (Fig. 4A). Given that no cell division occurs in oocytes up to ovulation, our findings indicated the preferential replication of the mutated haplotype independent of the cell cycle (relaxed replication (*22*) Fig. 4B) and negative selection against high heteroplasmy levels (Fig. 2D) (*23*). To test this, we developed six models of mtDNA replication in nondividing cells (Fig. 4C; see the “Modeling and statistical methods” section), which all assumed a fixed subset of mtDNAs replicating within an oocyte. The model that most closely aligned with experimental data (Fig. 4D) is consistent with the m.5024C>T haplotype having a replication advantage throughout development and a purifying selection against a level of heteroplasmy that depends on mtDNA copy number. Specifically, the cell can withstand a higher mutant heteroplasmy only if it contains a sufficient number of wild-type mtDNA molecules. We found that below a critical wild-type copy-number threshold (in the range of 10^2^ to 10^3^), we find that the cell can only tolerate a mutant heteroplasmy of 0.5 (SD = 0.2), and above the critical wild-type copy-number threshold, it can tolerate a larger mutant heteroplasmy of 0.8 (SD = 0.1). For a complete description of the model and interpretation, see the Supplementary Materials.

We then used our model to predict how germ cells would develop across the whole range of heteroplasmy values, and the effect this would have on the offspring (Fig. 4E). This indicated a net positive selection when m.5024C>T was below ~75% but a negative selection above this value. The modeling predictions based on mtDNA replication in oocytes matched our experimental observations seen during transmission of heteroplasmy from 42 mothers to 1167 pups (Fig. 1B). Last, we measured the mtDNA content, heteroplasmy shift (HS), and heteroplasmy variance in ovulated oocytes collected from two 7-week-old mothers (Fig. 4, F to I). This showed a slight reduction in mtDNA content compared to late antral oocytes, but a similar distribution of heteroplasmy values (mean heteroplasmy variance = 0.036). Although limited to two mothers with intermediate-high heteroplasmy values, this suggests that the process of ovulation itself does not alter the heteroplasmy values in oocytes. In keeping with this, the heteroplasmy variance in oocytes from antral follicles (Fig. 3E) was the same as the variance among offspring (pup’s mean heteroplasmy variance = 0.016; Fig. 1B). All of these findings are consistent with our observations during oocyte development being reflected in the offspring heteroplasmy levels (Fig. 1B).

### Potential mechanisms of selection during oocyte development

**Fig. 5.**
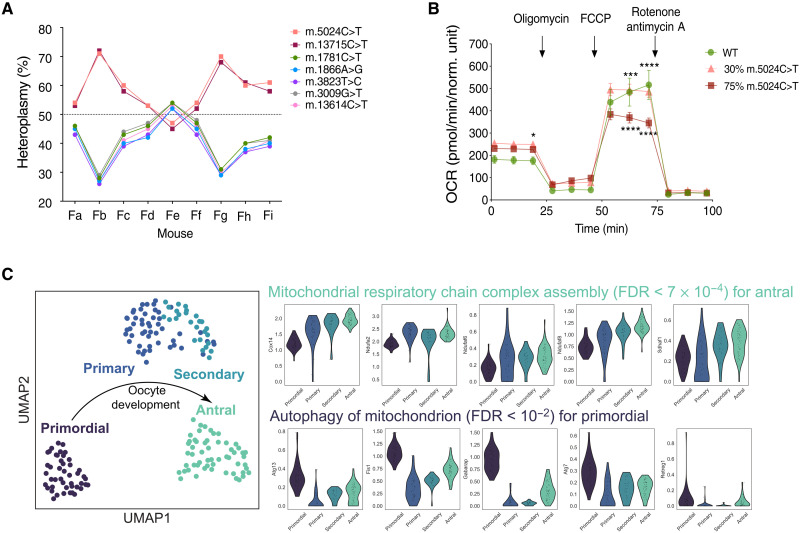
Potential mechanisms driving the mtDNA segregation. (**A**) Segregation of mtDNA variants in heteroplasmic *BVSC*-tRNA^Ala^ mice. Nine female (Fa to Fi) mice underwent whole mtDNA deep resequencing of genomic DNA extracted from an ear biopsy. The variant allele frequencies (heteroplasmy, %) for each mtDNA variant are linked with the same colored line to show two reciprocal mtDNA haplotypes, one including both the m.5024C>T and m.12715C>T variants. Average read depth (X = fold) per mtDNA position were as follows: Fa = 30,423X (SD = 15,902), Fb = 40,147X (SD = 20,395), Fc = 18,322X (SD = 8942), Fd = 44,367X (SD = 24,766.4), Fe = 128,836X (SD = 71,549.), Ff = 27,466X (SD = 13,952), Fg = 21,501X (SD = 12,017), Fh = 32,835X (SD = 15,995), and Fi = 8,293X (SD = 4,070.6). (**B**) Mitochondrial oxygen consumption rates (OCRs) of wild-type, 75%, and 30% m.5024C>T heteroplasmy fibroblast cell lines derived from C57BL/6NJ mice line. Data points show the mean from five independent measurements ± the SEM. The basal OCR at 20 min was marginally significantly different between the three cell lines (*P* = 0.0449) but not at the two earlier data points, making this unlikely to be biologically relevant. (**C**) Single-cell transcriptome analysis from oocytes at different stages of development reveals transcriptional changes during oocyte development [UMAP projection ((*48*)]. A GO term enrichment analysis reveals an overexpression of genes associated with “mitochondrial respiratory chain complex assembly” during the antral stage and of “autophagy of mitochondrion” during the primordial stage with multiple-testing correct FDRs of 7 × 10^−4^ and 1 × 10^−2^, respectively. We show gene expression for some selected example genes.

To determine whether additional mtDNA variants could explain the segregation, we deep resequenced ear biopsy mtDNA from nine mice. This confirmed that the m.5024C>T mutation cosegregated with the known linked variant (m.13715C>T/ND6) (*12*) and also confirmed the mouse strain (C57BL/6NJ). However, there were no other variants cosegregating with the mutant haplotype (Fig. 5A). Analysis of mouse embryonic fibroblasts harboring different heteroplasmy levels showed that high (>75%) heteroplasmy levels of the m.5024C>T mutation affected mitochondrial respiration (Fig. 5B), suggesting a lower biochemical threshold in bulk embryonic fibroblasts than seen in single cardiomyocytes (*13*), possibly reflecting known tissue-specific differences for mtDNA mutations. The maximal respiration rate in fibroblasts with low (30%) m.5024C>T levels was not different to fibroblasts with the background haplotype (zero m.5024C>T). Thus, the increase in m.5024C>T heteroplasmy from low levels is not being driven by a selection against an alternative haplotype through a respiration defect.

Last, we analyzed gene expression profiles from (*24*) for single oocytes at the four follicular stages we studied earlier: oocytes from primordial (P5), primary (P8 to P14), secondary (P15), and antral follicles (P20) (Fig. 5C). We observed a progressive increase in the expression of genes involved in “mitochondrial respiratory chain assembly” during oocyte maturation, and pathway analysis revealed a significant overexpression of these genes at the antral stage [false discovery rate (FDR) < 10^−3^], consistent with our direct measurements of mtDNA content (Fig. 4B). We also identified a significant overexpression of genes associated with “autophagy of the mitochondrion” during the primordial follicle stage oocyte (FDR < 10^−2^), which has been proposed as key mechanism involved in selfish mtDNA replication in invertebrates (*25*).

### No evidence of selection during sperm development and maturation

**Fig. 6.**
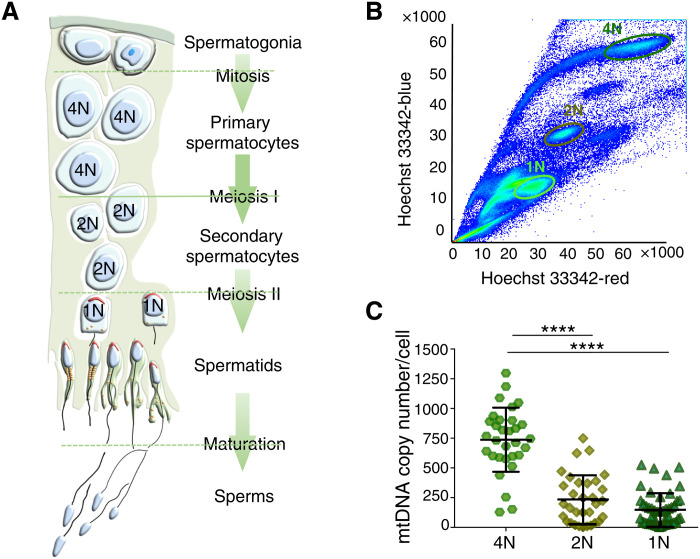
Isolation of single meiotic male germ cells from the *BVSC*-tRNA^Ala^ m.5024C>T mice. (**A**) Schematic representation of the major events during male spermatogenesis. *N* = number of matched nuclear chromosomes. (**B**) Florescence activated cell sort gating for 4N, primary spermatocytes, 2N secondary spermatocytes, and 1N spermatids. (**C**) mtDNA content in single meiotic male germ cells. ****P < 0.0001.

**Fig. 7.**
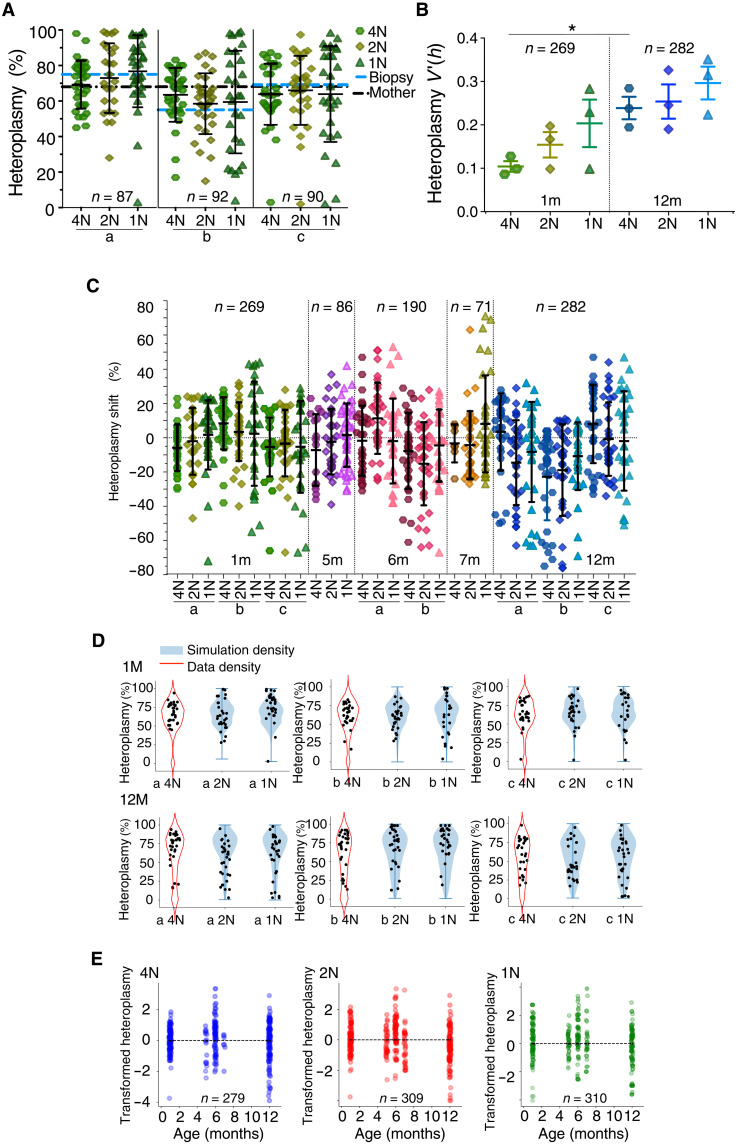
MtDNA analysis of single meiotic male germ cells in the *BVSC*-tRNA^Ala^ m.5024C>*T* Mice. N = number of matched nuclear chromosomes corresponding with Fig. 5 (A and B). (**A**) m.5024C>T heteroplasmy levels in single meiotic germ cells, matched male ear biopsy, and mother’s ear biopsy. Mouse a: biopsy = 68, mother = 75; mouse b: biopsy = 55, mother = 68; mouse c: biopsy = 69, mother = 68. Blue and black dashed horizontal lines correspond to the male ear biopsy and mother’s ear biopsy, respectively. (**B**) Normalized variance of mtDNA contents in single meiotic germ cell from mice of 1 to 12 months. *V*′(*h*) = *V*_h_/μ (1 − μ). *V*_h_ is heteroplasmy variance, and μ is mean heteroplasmy. *P = 0.05. (**C**) m.5024C>T heteroplasmy shift of single meiotic germ cell compared to the mother’s heteroplasmy level. Heteroplasmy shift = single germ cell heteroplasmy minus mother’s ear biopsy. a to c = different males at each age in months (m). (**D**) Simulation of male germ cell meiosis at the age of 1 and 12 months. Kernel density estimation was performed in mutant/wild-type copy number space on raw single-cell data (data density). Draws from the learned density were taken (*n* = 10,000 iterations), and one symmetric binomial partition step was applied per iteration for 2C and two partition steps for 1C cells. Violin plots display the simulation density. (**E**) Transformed m.5024C>T heteroplasmy level during male germ cell meiosis from the age of 1 to 12 months showing no change in the mean transformed heteroplasmy shift with age. Transformed heteroplasmy change = ln(*h*(*h*_0_ − 1)/*h*_0_(*h* − 1)). *h*, heteroplasmy.

Although mtDNA is not usually transmitted down the paternal line, we turned our attention to male gamete development to determine whether the mechanisms were specific to female germ line. Unlike oocyte development, during spermatogenesis, there are continuous cycles of both mitosis and meiosis, which generate millions of sperm throughout reproductive life (Fig. 6A). First, we established flow cytometric methods to isolate meiotic spermatocytes (*26*) (Fig. 6B and fig. S3) and measured the amount of mtDNA in 269 single cells from three 1-month-old mice (Fig. 6C) showing a sequential reduction of mtDNA content with each meiotic cell division (4N spermatocytes, mean mtDNA = 772.00, SD = 281.35; 1N spermatids, mean mtDNA = 91.81, SD = 92.40). Thus, the original pool of mtDNA molecules is sequentially partitioned during spermatogenesis, with no net increase in the total mtDNA content on cell division. During sperm development, the range and variance (Fig. 7, A and B) of heteroplasmy levels increased, in keeping with the vegetative segregation of alleles during cell division.

Unlike the late-stage oocytes, the mean heteroplasmy level throughout sperm development matched the ear biopsy from the same animal (Fig. 7A), and a simple model of vegetative segregation without selection reliably predicted the heteroplasmy distribution (Fig. 7D). Despite reaching extremely high levels (>95%), which are not seen in other organs (*12*), we saw no appreciable effect of selection against the mutation during sperm development (Fig. 7E). Extending our observations to 273 single cells from three 12-month-old mice, we saw an even greater range of heteroplasmy levels (Fig. 7C) and associated increased variance (Fig. 7B), with values ranging from 3 to 98% in spermatids from some mice. Again, there was no evidence of selection (Fig. 7, D and E, and table S3). Thus, the segregation of mtDNA heteroplasmy occurs not only during the 2 weeks of sperm meiosis (*27*) but also likely within the stem cell compartment throughout life. We explored the possible functional consequences of high heteroplasmy levels in mature sperm using in a 50 to 80% Percoll gradient but found no enrichment for low heteroplasmy levels in the high Percoll fractions (table S4), consistent with no physiological effect of the mutation in sperm. In conclusion, both the preferential replication of the mutant mtDNA haplotype and the purifying selection we observed are specific to the female germ line and are independent on cell division, indicating a subcellular mechanism acting at the organelle or mtDNA level.

### Comparison with heteroplasmy transmission in humans

**Fig. 8.**
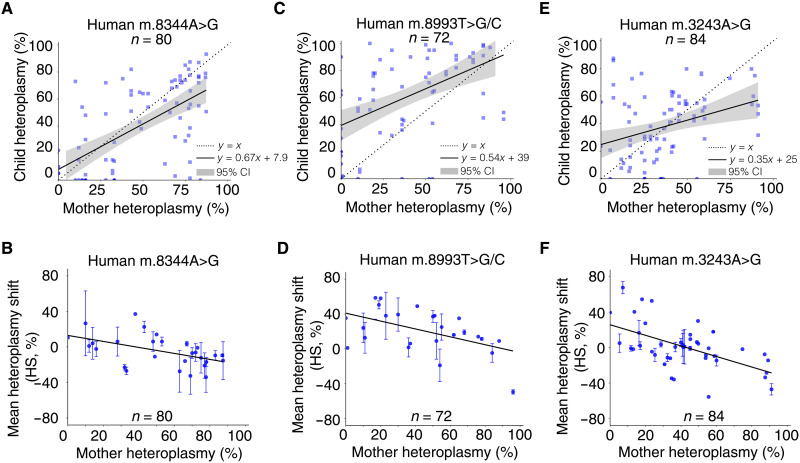
Transmission of pathogenic heteroplasmic mtDNA mutations in humans. Reanalysis of published data after minimizing ascertainment bias. Upper panels show the relationship between the maternal and offspring heteroplasmy levels. *y = x* corresponds to the 1:1 transmission of heteroplasmy from mother to her offspring. *y = mx + c* corresponds to the line of best fit for the data with 95% confidence intervals for the regression line (gray). Heteroplasmy levels were measured in blood from the human participants, and ear biopsies from the mice. Lower panels show the mean difference in heteroplasmy from an individual mother (HS). Error bars are SEM. (**A** and **B**) Human m.8344A>G mutation, from 34 mothers to 80 offspring. (**C** and **D**) Human m.8993T>G/C mutation, from 38 mothers to 72 offspring. (**E** and **F**) Human m.3243A>G.

We next looked for evidence that our observations had more general relevance. We compared our findings in mice to the transmission of mtDNA heteroplasmy in humans by reanalyzing 236 mother-child pairs ascertained from the clinic [Fig. 8, A to F; data from (*28*)]. Before plotting the data, we minimized the ascertainment bias by excluding the probands, as described (*28*). For several mutations, mothers with low heteroplasmy levels tended to have offspring with higher heteroplasmy levels, and mothers with high levels tended to have offspring with lower levels. This pattern resembled our findings in m.5024C>T tRNA^Ala^ mice (Fig. 1, B and C, and table S5) and corresponded to the model predictions (Fig. 4E) based on our analysis of single oocytes (Figs. 3 and 4). It is likely that the positive selection at lower heteroplasmy levels counterbalanced the negative selection at higher levels, explaining why the previous analysis (*28*) did not detect any selection overall.

## DISCUSSION

How do we explain the paradoxical replication bias favoring mutant mtDNA? Similar behavior has been observed in several invertebrate species, where a “selfish” replicator phenotype copies a deleterious mutation irrespective of the impact on the organism as a whole. In *Drosophila melanogaster*, temperature-sensitive variants in complex I confer a replicative advantage during oogenesis, despite the evidence of selection against the variant in somatic tissues during life (*29*). This resembles our findings in mice, where high levels of the m.5024C>T tRNA^Ala^ mutation compromise oxidative phosphorylation (*12*) but the mutant haplotype is preferentially replicated at lower heteroplasmy levels. In heteroplasmic *Caenorhabditis elegans*, the uaDf5 mtDNA haplotype escapes the conventional mechanism regulating mtDNA levels, leading to a wider variation in the amount of mutant mtDNA than wild-type mtDNA (*30*). We also saw a similar pattern (wild type, SD = 2.10 × 10^4^; mutant, SD = 3.02 × 10^4^), implicating a subtle difference in nuclear control of replication acting at the molecular level (*30*). Like the m.5024C>T tRNA^Ala^ mutation in mice, the uaDf5 genome in *C. elegans* also appears to be under opposing selective pressures, which have different strengths at different levels of heteroplasmy (*31*), possibly under nuclear genetic control (*4*). In several invertebrate species, the selfish drive most likely occurs at the level of the genome, rather than the organelle, cell, or organism (*25*). This is easy to understand when one molecule is smaller, but it can arise through single base substitutions, particularly in noncoding regulatory regions (*32*). As for temperature-sensitive *Drosophila* strains (*29*), we found no additional variants that could explain the propagation of the m.5024C>T haplotype.

A mechanism favoring the propagation of pathogenic mtDNA has been proposed for the m.8993T>G mutation in humans (*33*). Lymphocytes with high levels of m.8993T>G contain mitochondria with a high mitochondrial membrane potential (MMP), thought to arise because the mutation impairs proton flow through complex V (*34*). An increased MMP is required for oocyte maturation (*35*), and it has been suggested that mitochondria with a high MMP are recruited to the Balbiani body during oocyte development (*33*), where the mtDNA is preferentially replicated and transmitted to the offspring (*7*). Direct evidence supporting this mechanism is lacking at present but is consistent with our data. It remains to be seen whether other mutations behave in the same way. Alternatively, there could be an up-regulation of mtDNA replication within the mitochondria with impaired oxidative phosphorylation, resembling the “ragged red” response seen in skeletal muscle fibers harboring pathogenic mtDNA mutations (*36*), leading to an increase in the overall mutation load within individual oocytes.

Unlike sperm, we did not see late-stage oocytes containing high (>85%) heteroplasmy levels. This points toward a pre-ovulation mechanism contributing to the selection seen during transmission, defining a narrower time window than noted previously (*11*). Although this could be due to the loss of oocytes through an age-linked process such as atresia (*37*), we think this unlikely because the distribution of heteroplasmy levels in late antral oocytes from newborn mice was no different to the value in sexually mature mice. There are several alternative explanations for the purifying selection, including the loss of specific alleles at the subcellular level by mitophagy (*38*), which is linked to mitochondrial fission in the *Drosophila* germ line (*39*) or at the cellular level because of an effect on cellular fitness [reviewed in (*40*)]. However, it is difficult to understand how these would target a wild-type allele with no obvious biochemical effect. It will be challenging to explore these mechanisms because, as we have shown, the effects appear to be cell type and cell stage specific.

In conclusion, we have identified a complex pattern of heteroplasmy transmission in mice, and it is important to note that the mechanism we have uncovered may be specific to the m.5024C>T mtDNA mutation. The preferential propagation of mtDNA resembles the selfish replication seen in invertebrates and explains how disadvantageous low-level heteroplasmies increase during maternal transmission. It is conceivable that this explains why some pathogenic mtDNA mutations are more commonly seen in the clinic than others (*3*), as opposed to there necessarily being a difference in the base specific mutation rate. In keeping with this, the analysis of surplus oocytes, blastomeres, and zygotes from seven human mtDNA mutation carriers showed higher levels in the oocytes, blastomeres, and zygotes than the maternal blood (*33*). Selection against high levels of heteroplasmy will prevent the mutant genotype becoming fixed in the germ line, leading large heteroplasmic multigenerational pedigrees.

## MATERIALS AND METHODS

### Human heteroplasmy data

Mother-child heteroplasmy transmissions were analyzed in previously curated data derived from blood measurements made in pedigrees [citations and methods in (*28*)], incorporating age-corrected m.3243A>G values (*41*) and minimizing ascertainment bias through the exclusion of probands from the analysis. Ethical approval for this work was not required because the analysis only involved anonymized published data.

### Mouse husbandry

The *BVSC*-tRNA^Ala^ mice line was generated by crossing m.5024C>T single heteroplasmic mutant tRNAAla mice (*12*) with *BVSC* mice (*18*). DNA was extracted from ear biopsies for individual genotyping and mitochondrial heteroplasmy level analysis. All procedures conformed to the 1986 U.K. Home Office Animals Scientific Procedures Act (PPL P6C97520A) under license P6C97520A with approval from the University of Cambridge Animal Welfare Ethical Committee.

### Oocyte and embryo collection

Developing oocytes were manually isolated from primordial, primary, secondary, and antral follicles at P1, P8, P12, and P21 postnatal ovaries, respectively. Ear notch or tail biopsies were taken at the same time for DNA extraction. Isolated oocytes were observed under an epifluorescence microscope to confirm the diameters at desired developmental stage (*42*), as well as the morphology indicating quality. P1 ovaries were dissociated within collagenase and DNase I (Roche), stained with DRAQ7 (Abcam), and single cells were sorted using fluorescence-activated cell sorting (FACS) into 96-well polymerase chain reaction (PCR) plates. P8, P12, and P21 oocytes were manually picked from dissociated ovaries. For the collection of ovulated oocytes, mice underwent an intraperitoneal injection of 7.5 IU of equine chorionic gonadotropin (eCG) 48 hours before a human chorionic gonadotropin (hCG) injection. Fourteen to 16 hours after hCG priming, the oviducts were harvested and dissected. For zygote collection, superovulated females were put with males following hCG injection, and fallopian tubes were harvested and dissected 24 hours later. Retrieved oocytes/zygotes were denudated in 8 IU of hyaluronidase for 15 min and mechanically denudated by pipette thereafter. Oocytes/zygotes were washed three times in phosphate-buffered saline/0.1% bovine serum albumin, and then collected individually into 8-Strip PCR tubes. All samples were stored at −80°C until use.

### Spermatocyte FACS sorting and spermatozoa fractionation

The FACS protocol was adapted from published protocol (*26*), with minor modifications. Testes from male mice at the ages of 1, 3, 5, 6, 7, 9, and 12 months were dissected and decapsulated in L-15 medium and then washed in Hanks’ balanced salt solution without Ca^2+^ or Mg^2+^ twice. The seminiferous tubules were disrupted in TrypLE (Gibco) and then filtered twice through a 50-μm cell strainer (CellTrics) in 10% fetal bovine serum/Dulbecco’s Modified Eagle’s medium. After centrifugation, the pellet was resuspended in FACS solution and incubated for 45 min with Hoechst 33342 (Thermo Fisher Scientific) at the ratio of 0.5 μl of Hoechst 33342 to 1 × 10^6^ cells. DRAQ7 staining was carried out before single-cell FACS sorting into 96-well plates. Spermatozoa fractionation and Percoll (Sigma-Aldrich) gradient self-migration were performed as previously described (*43*), with minor modifications. Cauda epididymis was dissected in FERTIUP. Discontinuous Percoll gradient (50%-60%-70%-80%) was prepared with isotonic Percoll solution and EBSS (Earle’s Balanced Salt Solution) (Gibco) solution. One milliliter of sperm suspension was loaded onto layered discontinuous Percoll gradients and incubated at 37°C, 5% CO_2_ for 90 min. After self-migration, individual gradient layers were collected into tubes, diluted with 5 ml of EBSS, and then centrifuged. DNA from the resulting pellets were extracted with DNeasy Blood and Tissue Kit (Qiagen).

### Single-cell lysis

FACS-sorted or manually picked single cells were lysed with 0.5% Tween 20 (Sigma-Aldrich), 200 μM tris-HCl (pH 8.5), and proteinase K (1 mg/ml; Ambion) at 37°C for 30 min and then inactivated at 80°C for 15 min.

### Quantification of mtDNA copy number

Single-cell absolute mtDNA copy number was determined by droplet digital PCR targeting the mitochondrially encoded NADH (reduced form of nicotinamide adenine dinucleotide) dehydrogenase 1 (*MT-ND1*): GAGCCTCAAACTCCAAATA CTCACT (forward), GAACTGATAAAAGGATAATAGCTATGGTTACTTCA (reverse), and FAM-CCGTAGCCCAAACAAT (probe ); and mitochondrially encoded *Cytochrome C Oxidase III* (*MT-CO3*): CCTCGTACCAACACATGATCTAGG (forward), AGTGGGACTTCTAGAGGGTTAAGTG (reverse), and HEX-ACCTCCAACAGGAATTTCA (probe).

### Quantification of levels of heteroplasmy

Pyrosequencing was performed with PyroMark Q48 pyrosequencer (Qiagen) according to published protocol (*12*) using the following primers: /5biosg/TTCCACCCTAGCTATCATAAGC (forward), CGTAGGTTTAATTCCTGCCAATCT (reverse), and TGTAGGATGAAGTCTTACA (sequencing primer).

### Mitochondrial respiration measurements

Mitochondrial oxygen consumption rate (OCR) of wild-type, 30% and 75% mutant m.5024C>T mouse fibroblast lines was measured using a SeahorseXFe24 Analyzer. Final concentrations of 1 μM oligomycin, 1.5 μM FCCP, and 1 μM rotenone/1 μM antimycin A were applied sequentially. After extracellular flux analysis, cells were lysed in 30 μM lysis buffer and followed by Bradford protein assay. Seahorse data were normalized against protein level.

### Modeling and statistical methods

We simulated the stochastic replication of mutant (birth-rate λ*_m_*) and wild-type (birth-rate λ*_w_*) mtDNA within a population of nondividing cells, where a fixed subset (*R*_0_) of molecules replicate at any one time (analogous to there being a limited amount of replication machinery), and the number of mutant and wild-type molecules replicating is determined by the heteroplasmy level after each iteration. When the proportion of mutant mtDNA exceeded a threshold *h*_th_, further replication of mutant mtDNA is prevented. Notice that in the model with *H* = 5, this threshold value of heteroplasmy depends on the number of wild types in the cell: We call it *h*_th,1_ if *w* < *w*_th_, and *h*_th,2_ otherwise, where *h*_th,1_ < *h*_th,2_. An ABC rejection algorithm was performed to find posterior probabilities for the parameters λ*_m_*, λ*_w_*, *R*_0_, *h*_th_, *T*, *h*_th,1_, *h*_th,2_, and *w*_th_, and the indicator *H* corresponding to the six models:

1. *H* = 0: negative selection and λ*_m_*, λ*_w_* independent but constant throughout development (positive selection for any *t*);

2. *H* = 1: positive selection and with negative selection;

3. *H* = 2: negative selection and λ*_m_*, λ*_w_* set equal for *t* < *T* and independent for *t* > *T* (positive selection after *t* = *T*);

4. *H* = 3: no negative selection and positive selection after *t* = *T*;

5. *H* = 4: no negative selection and no positive selection;

6. *H* = 5: with positive selection throughout, but the threshold for negative selection depends on the number of wild-types: if *w* < *w*_th_, then *h* > *h*_th,1_$ for selection to switch on, whereas *h* > *h*_th,2_ if *w* > *w*_th_.

Statistical tests were performed on the transformed heteroplasmy (*20*) as follows

$$h^{\prime }=\text{ln}(\frac{h(1-h_{\text{pup}})}{h_{\text{pup}}(1-h)})$$

where *h* is the single-cell value of heteroplasmy and *h*_pup_ is an ear biopsy taken contemporaneously to the collection of oocytes. This definition of heteroplasmy has the advantage of showing the shift of the value *h* with respect to some initial value chosen (in this case, the aforementioned pup biopsy is the benchmark). Specifically, ℎ’ positive (negative) corresponds to larger (smaller) values of *h* compared to *h*_pup_. Given how it is constructed, its sign and magnitude contain information about the sign and the strength of any selection. Parametric statistical tests (one- and two-sample *t* tests) were used to compare the distributions, with all comparisons significant after Bonferroni correction for multiple possible comparisons (e.g., the eight possible thresholds between early and late oocyte development in fig. S3 and the five possible comparisons between PGCLCs in Fig. 4B).

### mtDNA sequencing and bioinformatics analysis

MtDNA was enriched with long-range PCR using PrimeSTAR GXL DNA Polymerase (R050B, Takara Bio) and the following primers: AGCAAAAGCCCACTTCGCCA (forward1), GGTTGGCCCCCAATTCAGGT (reverse1), ACCTGAATTGGGGGCCAACC (forward2), and TGGCGAAGTGGGCTTTTGCT (reverse2). Amplicons were purified with AMPure XP (A63880, Beckman Coulter), quantified using Qubit 2.0 fluorometer (Q32853, Thermo Fisher Scientific) and Qubit assay tubes (Q32856), fragmented using a NEBNext Ultra II FS DNA Library Prep Kit, followed by adaptor ligation using the NEBNext Ultra II Ligation Module. Sequencing was performed on an Illumina Miseq using an NEBNext Ultra II Q5 Master Mix and NEBNext Multiplex Oligos. Fastq files were checked for sequence quality using FastQC (www.bioinformatics.babraham.ac.uk/projects/fastqc/). Trim Galore! (www.bioinformatics.babraham.ac.uk/projects/trim_galore/) was used to remove low-quality ends from reads (with quality score < 20) in addition to adapter removal. Reads shorter than 35 bp after trimming were removed. MtDNA variant calling was performed using a modified version of the MToolBox pipeline (*44*), which supports the analysis of nonhuman mtDNA (https://github.com/clody23/MToolBox-Ark/tree/noAdapter). Briefly, the GRCm38 mouse build (mm10) was used as reference genome for read mapping including the mtDNA reference sequence of the C57BL/6J mouse strain. A simultaneous mapping of reads onto nuclear and mitochondrial reference genomes was performed to remove ambiguously mapped reads derived from nuclear-mitochondrial sequences (NumtS). Removal of PCR duplicated was performed with MarkDuplicates (https://broadinstitute.github.io/picard/command-line-overview.html#MarkDuplicates), with a step integrated in the MToolBox workflow. Variant calling was performed with MToolBox default parameters (i.e., quality score ≥ 25; number of reads supporting the alternative variant ≥ 5; variants within five nucleotides from reads ends removed), and heteroplasmy fractions (%) were calculated as ratio between the number of reads supporting the variant allele and the total number of reads supporting the variant position. Oocyte transcriptome data were reanalyzed from a published dataset (*24*), based on 5877 oocytes collected from C57BL/6Babr mice. RNA was extracted and sequenced at the following stages: P5, oocytes *n* = 1545, raw sequencing reads = 44,111,984; P8 to 14, oocytes *n* = 1990, raw sequencing reads = 67,882,721; P15, oocytes *n* = 1510, raw sequencing reads = 118,463,451; and P20, oocytes *n* = 832, raw sequencing reads = 47,855,997. The gene ontology (GO) term gene lists were selected from the Mouse Genome Informatics website www.informatics.jax.org/tools.shtml.

### Kimura distribution analysis

Kimura distributions and cumulative curves were performed as described (*19*, *20*). Kolmogorov-Smirnov analysis was used to compare the observed mtDNA heteroplasmy data to a theoretical Kimura probability distribution.

### Single-cell gene expression analysis

We analyzed publicly available single-cell transcriptomics data (GSE114822) (*45*). For the expression matrix analysis, we used Scanpy (*46*) and performed standard preprocessing steps: removing cells with less than 200 genes, removing genes that have been detected in less than three cells, normalization to 10,000 reads per cell, and log transformation. Wilcoxon rank-sum tests were used with significance threshold of 0.05 for DEG (Differentially Expressed Genes) discovery for each oocyte maturation stage, and we used the Benjamini-Hochberg procedure to obtain multiple testing–corrected *P* values. To identify enriched GO, we used PANTHER (*47*) with the Benjamini-Hochberg procedure to obtain multiple testing–corrected FDR.

## Supplementary Materials

### This PDF file includes:

Figs. S1 to S11 Tables S1 to S5 References

### Other Supplementary Material for this manuscript includes the following:

Source Data Excel file

## Notes

View/request a protocol for this paper from *Bio-protocol*.
