# Supplementary material for: Mitochondrial DNA heteroplasmy is modulated during oocyte development propagating mutation transmission
Source: Sci Adv. 2021 Dec 8;7(50):eabi5657. doi: 10.1126/sciadv.abi5657 (PMC8654302; doi:10.1126/sciadv.abi5657)
Supplement: Supplementary file 1 — Figs. S1 to S11 Tables S1 to S5 References [file sciadv.abi5657_sm.pdf]

Science Advances
AAAS

Supplementary Materials for

Mitochondrial DNA heteroplasmy is modulated during oocyte development propagating mutation transmission

Haixin Zhang, Marco Esposito, Mikael G. Pezet, Juvid Aryaman, Wei Wei, Florian Klimm, Claudia Calabrese, Stephen P. Burr, Carolina H. Macabelli, Carlo Viscomi, Mitinori Saitou, Marcos R. Chiaratti, James B. Stewart, Nick Jones, Patrick F. Chinnery*

*Corresponding author. Email: pfc25@cam.ac.uk

Published 8 December 2021, *Sci. Adv.* **7**, eabi5657 (2021)
DOI: 10.1126/sciadv.abi5657

This PDF file includes:

Source Data Excel file
Figs. S1 to S11
Tables S1 to S5
References

Source Data Excel File
Contains the raw data used in main Figures 1-8 and Supplementary Fig.S3A

Fig. S1.

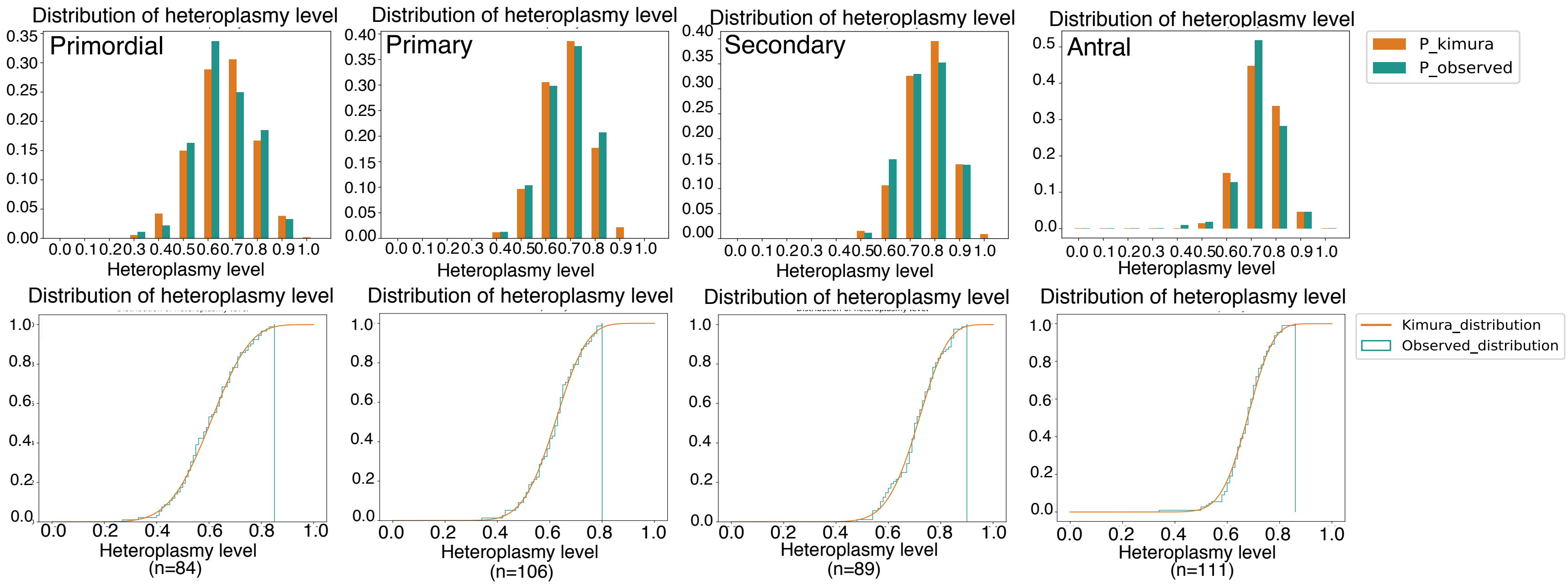

Supplementary Fig. 1. Kimura distribution and cumulative curve simulation of single cell mtDNA heteroplasmy dynamics during oocyte development in the BVSC-tRNA^{Ala} m.5024C>T mice. P1 Primordial follicle oocyte, n=84. P8 Primary follicle oocyte, n=106. P12 Secondary follicle oocyte, n=89. P21 Antral follicle oocyte, n=111. Upper panel shows the predicted Kimura distribution and observed distribution. Lower panel shows a comparison of the cumulative Kimura and observed distributions. Statistical analysis shown in Table S1.

Fig. S2.

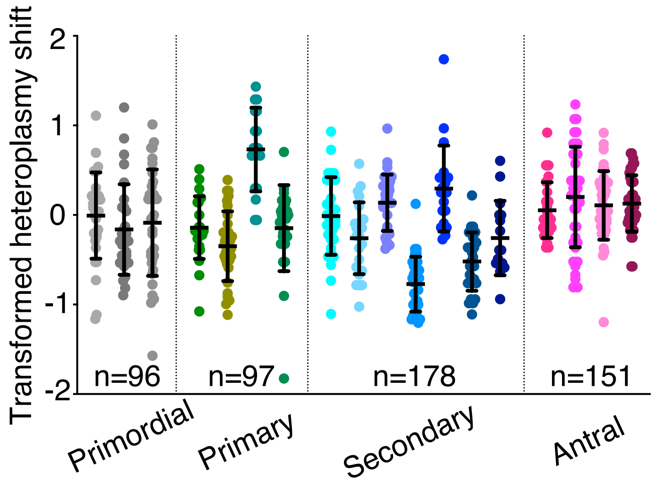

Supplementary Fig. 2. Transformed m.5024C>T heteroplasmy shift during oocyte development in the BVSC-tRNA^{Ala} m.5024C>T mice. Transformed m.5024C>T heteroplasmy shift during oocyte development = $\ln(h(h_0-1)/h_0(h-1))$. h, oocyte heteroplasmy measurement, oocyte or pup. h_0 = corresponding mother's ear biopsy. P1 Primordial follicle oocyte, n=96. P8 Primary follicle oocyte, n=97. P12 Secondary follicle oocyte, n=178. P21 Antral follicle oocyte, n=151.

Fig. S3.

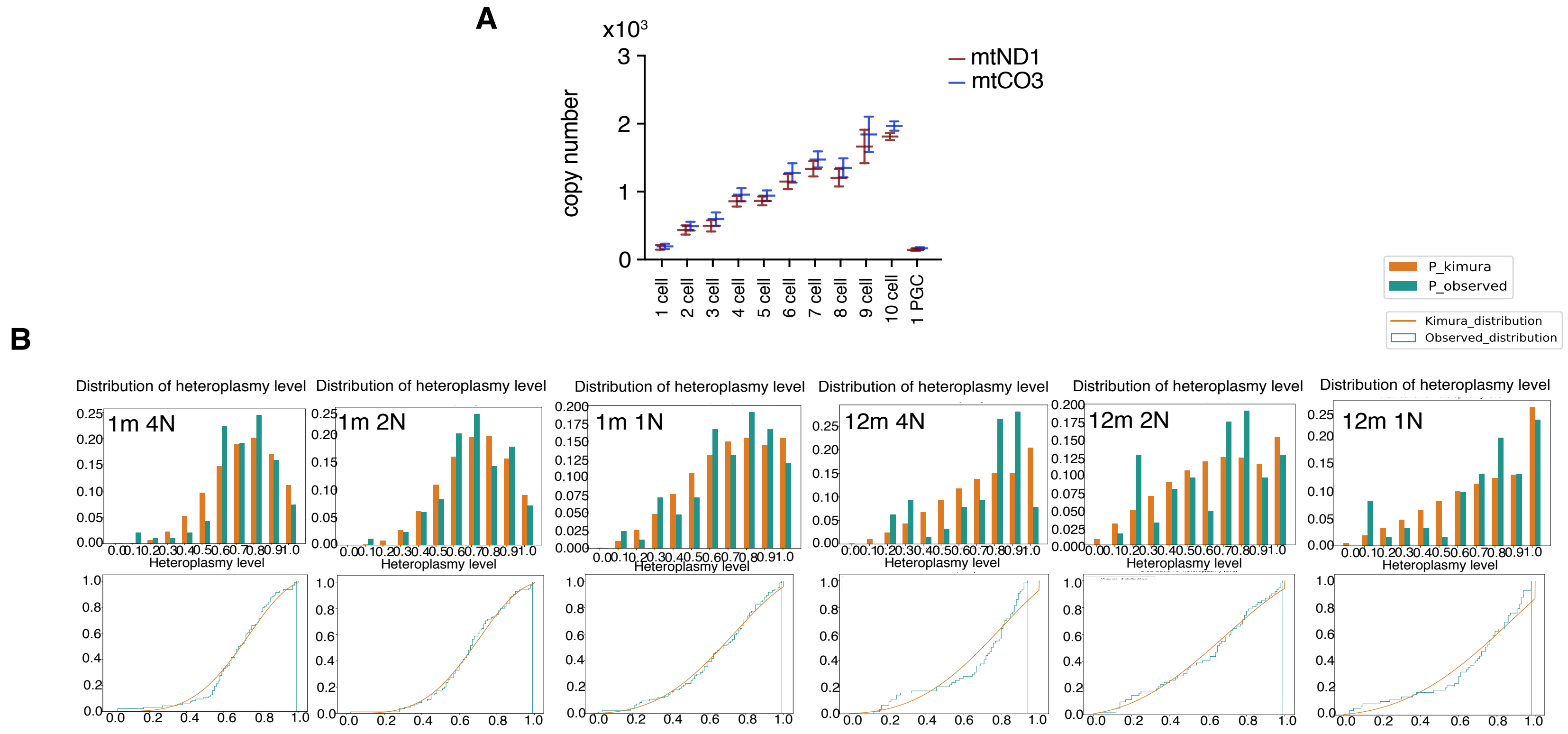

Supplementary Fig. 3. Single cell sorting and digital droplet PCR technique validation and Kimura analysis of mitochondrial heteroplasmy levels during male germ cell meiosis in the BVSC-tRNAAla m.5024C>T mice. (A) mtDNA copy number measured using independent assays against the mtND1 and mtND3 genes in FACS sorted cells, showing a linear relationship between the number of cells (1 to 10) and the mtDNA copy number measurement, thus providing a technical validation of the assay. PGC = primordial germ cell. Bars show the mean ± SD. **(B)** Kimura distribution and cumulative curve of mitochondrial heterplasmic levels in single meiotic male germ cells. 1month 4N spermatocytes, n=94. 2N spermatocytes, n=82. 1N spermatids, n=84. 12 month 4N spermatocytes, n=92. 2N spermatocytes, n=91. 1N spermatids, n=90. Upper panel shows the predicted Kimura distribution and observed distribution. Lower panel shows a comparison of the cumulative Kimura and observed distributions. Statistical analysis shown in Table S3.

Table S1.

Kolmogorov-Smirnov test of the Kimura distribution simulation and cumulative curve for single mouse oocytes. Analysis corresponds to Fig. S1.

Female	Age	Developmental stage	P value, Kolmogorov–Smirnov test
P1a	P1	Primordial follicle	0.798
P1b	P1	Primordial follicle	0.524
P1c	P1	Primordial follicle	0.493
P8a	P8	Primary follicle	0.602
P8b	P8	Primary follicle	0.858
P8c	P9	Primary follicle	0.83
P12a	P12	Secondary follicle	0.842
P12b	P12	Secondary follicle	0.825
P12c	P13	Secondary follicle	0.593
P21a	P21	Antral follicle	0.128
P21b	P22	Antral follicle	0.803
P21c	P22	Antral follicle	0.933
Merge	P1	Primordial follicle	0.802
Merge	P8	Primary follicle	0.822
Merge	P12	Secondary follicle	0.637
Merge	P21	Antral follicle	0.806

Table S2

Age and ear biopsy heteroplasmy values for the mothers and pups corresponding to Figure 2.

Female	P1a	P1b	P1c	P8a	P8b	P8c	P9	P12a	P12b	P13a	P13b	P13c	P14a	P14b	P21a	P21b	P22a	P22b
Age	P1	P1	P1	P8	P8	P8	P9	P12	P12	P13	P13	P13	P14	P14	P21	P21	P22	P22
Ear biopsy heteroplasmy (%)	60	63	64	68	73	76	56	78	72	73	79	65	82	80	71	82	63	64
Mother ear biopsy heteroplasmy (%)	67	62	62	61	77	70	61	76	61	77	70	70	70	70	67	70	63	63

Table S3.

Kolmogorov-Smirnov test of the Kimura distribution simulation and cumulative curve of single meiotic cells isolated from male mice. Analysis corresponds to Fig. S3. m=months

Male	Age	Developmental stage	P value, Kolmogorov–Smirnov test
1Ma	1m	4N	0.577
		2N	0.983
		1N	0.769
1Mb	1m	4N	0.775
		2N	0.924
		1N	0.974
1Mc	1m	4N	0.368
		2N	0.827
		1N	0.811
12Ma	12m	4N	0.328
		2N	0.766
		1N	0.488
12Mb	12M	4N	0.283
		2N	0.501
		1N	0.395
Merge	1m	4N	0.256
	1m	2N	0.78
	1m	1N	0.977
Merge	12m	4N	0.063
	12m	2N	0.62
	12m	1N	0.125
1Ma	1m	Merge	0.734
1Mb	1m	Merge	0.497
1Mc	1m	Merge	0.48
12Ma	12m	Merge	0.219
12Mb	12m	Merge	0.058

Table S4.

Fractionation of sperm in discontinuous Percoll gradients from m.5024C>T males. Mean heteroplasmy value (%) for each Percoll sperm fraction.

**Male**	**M6939**	**M6940**	**M1629**	**M1631**	**M1632**	**M1647**
Age (month)	9m	9m	2m	2m	2m	2m
Ear biopsy heteroplasmy (%)	63%	40%	79%	73%	77%	75%

Table S5.

Linear regression and correlation analysis of heteroplasmy transmission in humans and mice. M = gradient, c = Y-intercept. p-value is for the regression equation.

**Child/Pup-Mother**	**m**	**c**	**p-value**	**R^2**
**Human m.8344A>G**	0.67	7.9	4.576e-09	0.35
**Human m.8993T>G/C**	0.54	39	8.746e-06	0.24
**Human m.3243A>G**	0.35	25	0.002408	0.10
**Mouse m.5024T>C**	0.65	21	2.2e-16	0.44

1. Statistical tests

Statistical tests were performed on the transformed heteroplasmy data. The scope was to check whether there was a statistically significant difference in the heteroplasmy distributions over time. Transformed heteroplasmy is defined as [21]

$$h' = \ln\left(\frac{h(1-h_{\text{pup}})}{h_{\text{pup}}(1-h)}\right), \tag{1}$$

where h is the single cell value of heteroplasmy and h_{pup} is an ear biopsy taken contemporaneously to the collection of oocytes. This definition of heteroplasmy has the advantage of showing the shift of the value h with respect to some initial value chosen (in this case, the aforementioned pup biopsy is the benchmark). Specifically, h' positive (negative) corresponds to larger (smaller) values of h compared to h_{pup}. Given how it is constructed, its sign and magnitude contain information about the sign and the strength of the selection.

We designated all pre-antral cells as early and all antral cells late. We used a one-sample one-tailed t-test to explore shifts from zero: this allows us to reject the hypothesis that there is no-negative selection acting on the early cells. We used a two-sample one-tailed t-test to explore positive selection between the early and late cells. The tests assume a particular time as distinctive from the others: there are between 4 and 8 possible times we could have selected as distinctive. Both results remain significant after Bonferroni correction for 8 hypotheses.

2. Details of model and inference

2.1. Birth model

The dynamics of the system is simulated through a pure Poisson birth process encompassing the following reactions for mutants (m_{rs}) and wild-types (w_{rs})

$$m_{rs} \xrightarrow{\lambda_m} m_{rs} + 1 \tag{2a}$$

$$w_{rs} \xrightarrow{\lambda_w} w_{rs} + 1 \tag{2b}$$

Importantly, the replicating set (to which the subscript rs refers) of molecules does not coincide with the whole content of the cell, but is restricted to a

subset of fixed size $R_0 = m_{rs} + w_{rs}$, whose composition (the proportion m_{rs} to w_{rs}) varies in time. The ratio of m_{rs} to w_{rs} is defined to be the same as that in the whole cell: it is recomputed after each birth event. The idea of a fixed replicating subpopulation is implied in the immigration-like structure in [49] and yields a model with an approximately linear growth in population size (as is observed in the copy-number data). When cell heteroplasmy h exceeds a set threshold h_{th}, the birth-rate of mutants is set to zero, while wild-types continue to reproduce at a rate λ_w. This is equivalent to introducing a mechanism of negative selection against mutants: this might be because, above the threshold, the replication is impeded or a process like selective mitophagy exists. If the total cellular mtDNA population is below R_0 we suppose that the dynamics is that of a conventional birth process.

In one of our models we suppose that a selective advantage only exists after a time T: before this time $\lambda_m = \lambda_w$, after this time they are free to take distinct values.

Also, in one of the models we consider that negative selection switches on at a value of the heteroplasmy that depends on the number of wild-types - specifically, at h_{th1} if $w < w_{th}$, and at $h_{th2} > h_{th1}$ if $w > w_{th}$. This is consistent with Refs. including [16, 51].

The parameters of the most general model are $\lambda_m, \lambda_w, R_0, h_{th1}, h_{th2}, w_{th}, T$ (and H, a categorical parameter denoting which of six models is chosen, see below). For a given parametrization, we simulate the model using the Gillespie algorithm, also known as the stochastic simulation algorithm [50], where one stochastic trajectory corresponds to one cell in the experimental dataset.

2.2. Initial conditions

The experiment runs from day 1 to day 22 after the birth of the pup, so time will henceforth be measured in days post birth (dpb). Initial conditions for our model are the initial amount of mtDNA present in the cell n_0 and the initial heteroplasmy of the cell h_0 at day 1, where the heteroplasmy is defined as $h = m/(m + w)$. Each measured cell can have a distinct initial condition – as inferred from the ear-biopsy of the corresponding pup.

The initial copy number is drawn from a Gaussian kernel density estimation using the data from the three distributions of the day 1 (corresponding to the three pups that contributed the oocytes). The distribution was truncated with a lower bound of $n_{\min} = 75$, which is the minimum value of the day 1

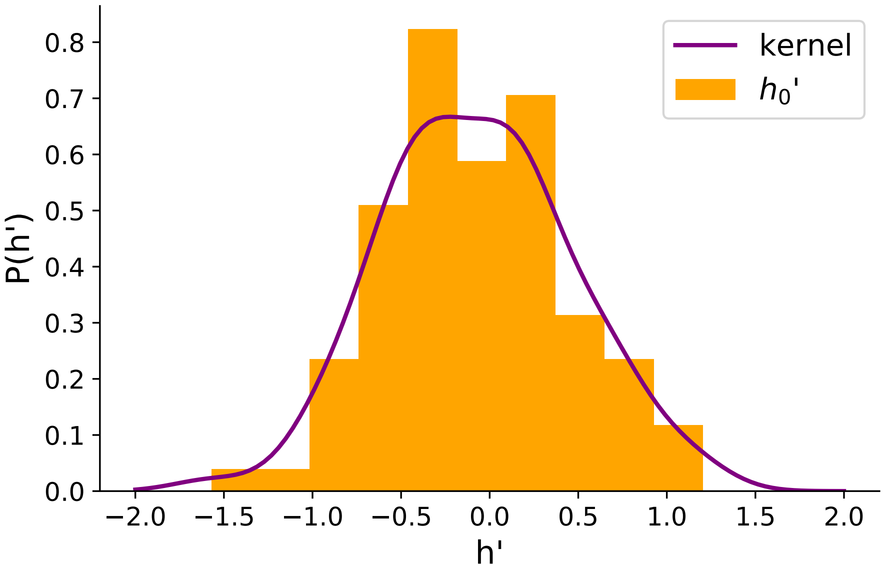

Figure S4: Day 1 transformed heteroplasmy h'_0 (orange) and Gaussian kernel fit to h'_0 (purple).

copy number data.

Our inference requires an initial heteroplasmy for each oocyte at day 1. We, instead, have knowledge of the ear-biopsy data for the pup from which the oocytes are removed. This ear-biopsy state is supposed to be the heteroplasmy value at an earlier time than day 1. We thus needed a model that takes as its input the pup ear biopsy for each pup and outputs a suitable candidate for its initial heteroplasmy at day 1. In order to build such a model, we assumed that the data that we already have from pups at day 1 contains the information about how selection acts on the initial (ear) heteroplasmy up to day 1. We thus first pooled together the day 1 transformed heteroplasmy distributions for each of the three pups obtaining h'_0. Then, we performed kernel density estimation (using a Gaussian kernel) to h'_0 as shown in Fig.S4. The estimated density (violet in Fig. S4) serves as a baseline to obtain initial values for the transformed heteroplasmy. For pup i we can draw from the estimated density and build a vector $\mathbf{h}^i_{\text{ker}}$ with as many elements as the number of oocytes said pup contributed. Inverting (1) we can finally determine the

initial heteroplasmy of oocyte j, pup i

$$h^i_{0,j} = \frac{h^i_{\text{pup}} e^{h^i_{\text{ker, j}}}}{1 - h^i_{\text{pup}}(1 - e^{h^i_{\text{ker, j}}})} \tag{3}$$

where h^i_{pup} is the value of the pup ear biopsy.

In all instances the Gaussian kernel has been found the Scott's method to estimate kernel bandwidth.

An alternative approach is to simply suppose that the initial heteroplasmy of each oocyte at day 1 corresponds to the ear-biopsy heteroplasmy. Doing this in fact leads to very similar results.

2.3. Inference

Inference of the model parameters $H, \lambda_m, \lambda_w, R_0$, T, $h_{th,1}$, $h_{th,2}$ and w_{th} was performed using the ABC rejection algorithm. H is an indicator parameter which encodes which model is being considered. We explored the following options:

- $H = 0$, with positive and negative selection;
- $H = 1$, with negative and without positive selection;
- $H = 2$, with negative selection and positive selection starting at time T during development, whereas $\lambda_m = \lambda_w$ before T;
- $H = 3$, without negative selection and positive selection starting at time T;
- $H = 4$, where neither positive nor negative selection are present;
- $H = 5$, with positive selection throughout, but negative selection depends on the number of wild types: selection switches on at $h > h_{th,1}$ if $w < w_{th}$ $h > h_{th,1}$, at $h > h_{th,2}$ if $w > w_{th}$.

Uniform priors are used for the heteroplasmy mitophagy thresholds h_{th} and $h_{th,2} \sim U(0,1)$, whereas the prior for h_{th1} is $U(0, h_{th2})$. A log uniform distribution is chosen for the two replicative rates $\log_{10} \lambda_i \sim U(1,3)$, $i \in \{m, w\}$, replicative subset size $\log_{10}(R_0) \sim U(1,3)$, and the threshold number of wild types $\log_{10}(w_{min}) \sim U(1,5)$ as we are unclear what order of magnitude

to expect. Our prior for H is such that $p(H = 0) = p(H = 1) = p(H = 2) = p(H = 3) = p(H = 4) = p(H = 5) = 1/6$, (this corresponds to five equally weighted priors for all the models we have considered: the experiment runs for 22 days in total).
The distance metric $\rho(\mathcal{D}, \mathcal{D}')$ chosen measures the absolute values between the simulated $\mathcal{D}'$ and the experimental dataset $\mathcal{D}$

$$\rho(\mathcal{D}, \mathcal{D}') = \sum_{i=1}^{15} A_0 \left|\mathbb{E}_{\mathcal{D}}(h')_i - \mathbb{E}_{\mathcal{D}'}(h')_i\right| + A_1 \left|\mathbb{E}_{\mathcal{D}}(n)_i - \mathbb{E}_{\mathcal{D}'}(n)_i\right| + A_2 \left|\mathbb{V}_{\mathcal{D}}(h)_i - \mathbb{V}_{\mathcal{D}'}(h)_i\right| \tag{4}$$

where $\mathbb{E}(h')$ is the mean transformed heteroplasmy, $\mathbb{E}(n)$ is the mean copy number, $\mathbb{V}(h)$ is the heteroplasmy variance and the subscript specifies whether the statistics are calculated on the experimental ($\mathcal{D}$) or simulated dataset ($\mathcal{D}'$). We sum over all the pups except for the pups at $t = 1$ dpb which were used for setting the model's initial conditions. A_j, $j = 0, 1, 2$, are normalization constants used to weight the three contributions approximately equally. Specifically, the summary statistics were normalized by the range of the corresponding experimental data. These are $A_0^{-1} = 1.5, A_1^{-1} = 1.56 \times 10^5, A_2^{-1} = 1.47 \times 10^{-2}$. We drew 6×10^6 samples from our prior for the ABC rejection algorithm.

The parametrizations were ranked according to their corresponding value of the distance metric $\rho(\mathcal{D}, \mathcal{D}')$, a smaller value of the latter corresponding to a stricter agreement to of the former to the data. We then looked at which model was more represented upon considering decreasing values of the distance metric, as a smaller threshold forces a stricter agreement with experimental data. As Fig. 2d (main text) shows, the model that best fits the data is $H = 5$, where negative selection depends on a threshold number of wild types. The best 200 parametrizations from model $H = 5$ have been retained as posterior distributions.

It is interesting to look at why the $H = 5$ model describes the data better than the others. If we look at the three error statistics we have considered (Fig. S5, data in red) we see that transformed heteroplasmy (panel (a)) dynamics seems to be split in two different phases: an early one and a later one, with a splitting point at some time in the range $14 - 20$. During the early stage there is no evidence for positive selection, but rather h' undergoes a slight decrease, whereas the latest points at days $21 - 22$ show that h' is trending upward. Similarly, the heteroplasmy variance $V(h)$ (panel (c)) shows

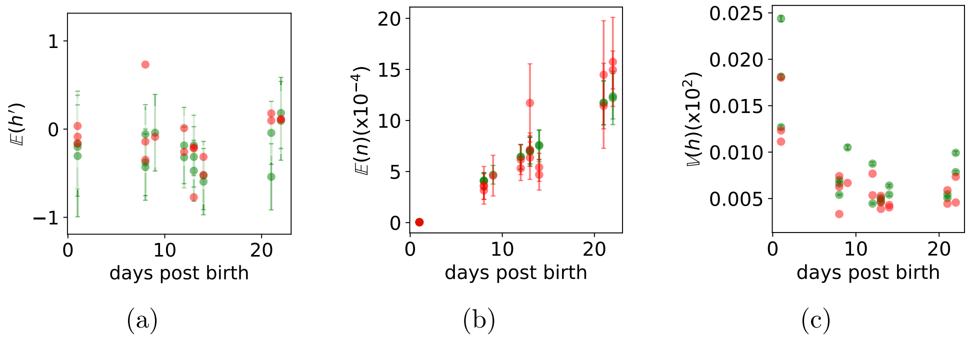

Figure S5: Mean posterior predictive distributions for the summary statistics considered in the ABC (red is the data and green is simulations).

a decrease after day 1. Models $H = 2$ and $H = 5$ are the best equipped to capture both phenomena (see discussion in sec 3 for model 5). Indeed, model $H = 1$ would not explain the decrease in $V(h)$, whereas model $H = 0$ cannot capture the two-stage dynamics of transformed heteroplasmy. Also, Looking at Fig. 3d (main text) we see that the worst performing models are those without negative selection ($H = 3$, $H = 4$). The poor ranking of these two models is likely due to there being very few data points with high heteroplasmy ($h > 0.9$): a feature that points to the existence of a purifying mechanism against very high levels of mutations (e. g. a negative selection like the one considered). To sum up, models 2 and 5 are both valid candidates in order to describe the trends in experimental data, but 5 performs better than the other one, and it is also more plausible from a biological standpoint [16, 51].

In Figs. S6 and S7, we display approximate posterior distributions of the parameters from ABC for model $H = 5$. Small values for the size of the replicative subset R_0 (compared to population size of the cell) are favoured (Fig. S6c), because the exponential growth that the model undergoes until it reaches $n = R_0$ is incompatible with the linear copy number growth observed in the data. The posteriors of the birth rates λ_m, λ_w are better understood by looking at their covariation with R_0 (Fig. S8b,c) that clearly show the trade-off R_0 and their magnitude. This reflects the fact that the products $R_0\lambda_m$ and $R_0\lambda_w$ are peaked (mean $\pm$ standard deviation $6.3 \cdot 10^3 \pm 1.1 \cdot 10^3$; $5.5 \cdot 10^3 \pm 9.8 \cdot 10^2$, Fig. S10a,b) because of the constraint due to copy number data that shows a clear linear trend (Fig. S5b). From the posteriors of the

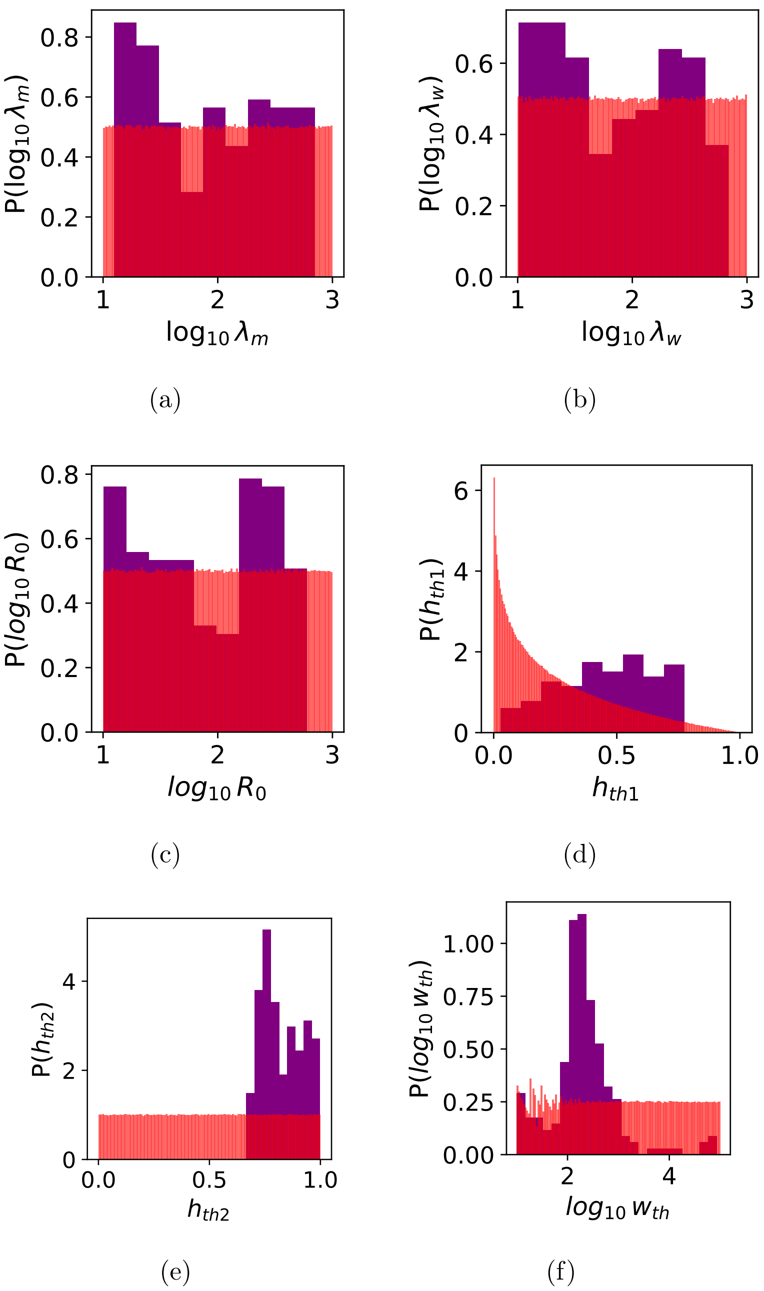

Figure S6: Posteriors of the $H = 5$ model parameters found using ABC.

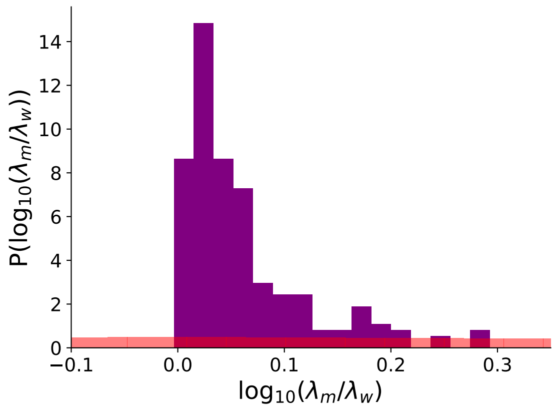

Figure S7: Posterior for the model $H = 5$ of the log mutants replicative advantage $\log_{10}(\lambda_m/\lambda_w)$. The model supports λ_m being 1.1 ± 0.18 (median $\pm$ standard deviation) larger than λ_w.

heteroplasmy thresholds h_{th1}, h_{th2} (Fig. S6d,e) (mean $\pm$ standard deviation $0.45 \pm 0.20; 0.82 \pm 0.1$, respectively) it is clear that the negative selection is needed for the model to agree with the data (see discussion in sec. 3 for more details on this aspect). The distribution of the threshold wild-types value w_{th} (Fig. S6f) at which negative selection changed intensity has the most support for values in the range $10^2 - 10^3$, which is early on in the dynamics. Lastly, since the relative advantage $\lambda_m/\lambda_w > 1$, the model fully supports mutants replicating faster than wild-types throughout the process ($P = 99.5\%$), as expected.

To sum up, posteriors point to a model ($H = 5$) where below a moderate-sized wild-type copy-number ($w_{th} = 10^2 - 10^3$) the heteroplasmy threshold for tolerating mutants is low ($h_{th1} \sim 0.5$) whereas above the characteristic wild-type copy-number the cells become more tolerant of mutant ($h_{th2} \sim 0.8$). The implication of this is that early in development, when copy-number is smaller, cells with higher initial heteroplasmy experience strong negative selection. This serves to pull down average heteroplasmy early in development. Later in development this negative selection is weaker (because the wild-types' copy-number is above w_{th}) allowing the continuous mutant positive selection to push up mutant-copy-number.

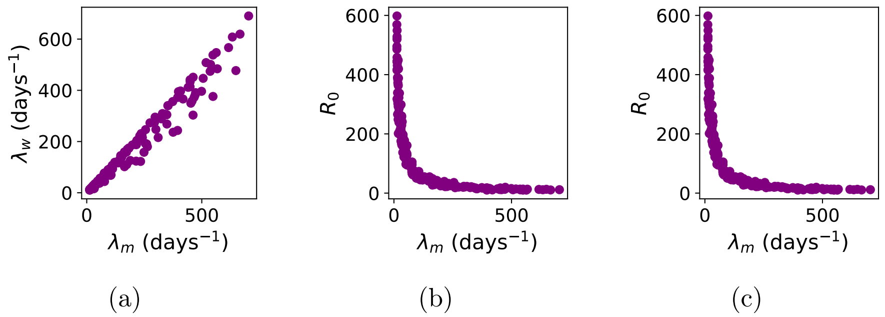

Figure S8: Bivariate plots of some of the $H = 5$ model parameters. Others did not show strong covariation and are not showed here.

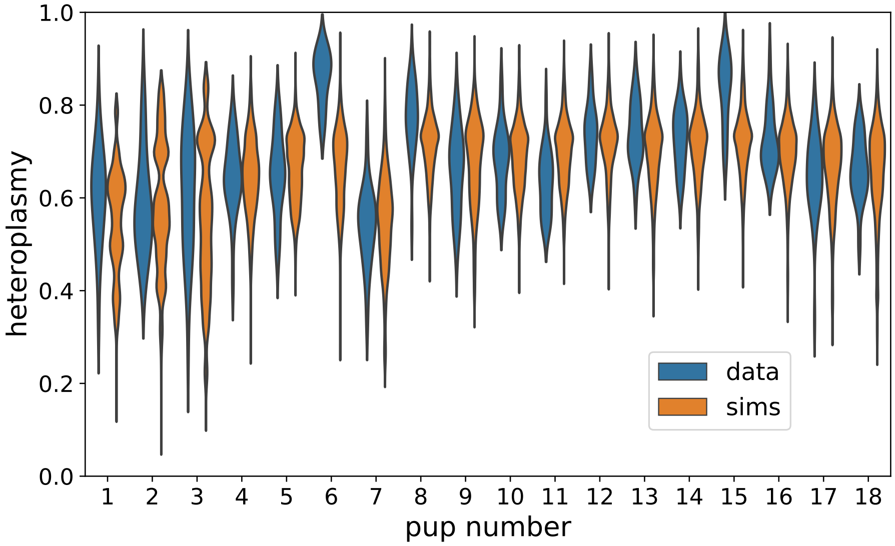

Figure S9: Heteroplasmy posterior predictive distributions (orange) compared to experimental data (blue) for the 18 pups.

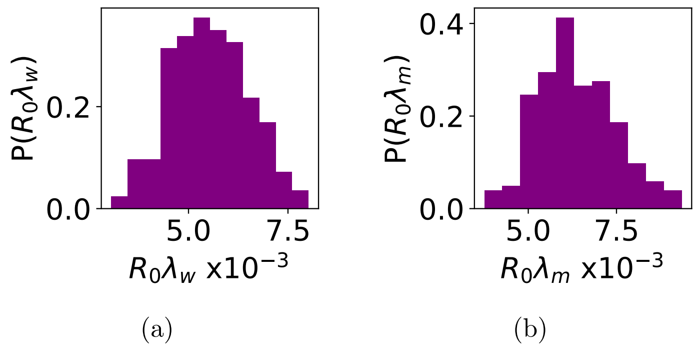

Figure S10: Posterior of the products $R_0\,\lambda_w$ and $R_0\,\lambda_m$.

3. Analysis of a deterministic variant of the model

In order to better understand the dynamics of the chosen model ($H = 5$), we considered one of its deterministic variants. In this context the dynamics is described by

$$\begin{aligned}\dot{m} = \;& \lambda_m m \theta(R_0 - n) + \\ & \lambda_m R_0 h[\theta(h_{th,1} - h)\theta(w_{th} - w) + \\ & \theta(h_{th,2} - h)\theta(w - w_{th})]\theta(n - R_0)\end{aligned} \tag{5}$$

$$\begin{aligned}\dot{w} = \;& \lambda_w w \theta(R_0 - n) + \\ & \lambda_w R_0 (1 - h)\theta(n - R_0)\end{aligned} \tag{6}$$

Equations (5) and (6) describe an exponential growth of mutants and wild-types until a time t^* when their total number equals R_0, and a quasi-linear growth from t^* onward. The two species replicate at their own rate λ_m and λ_w for mutants and wild types, respectively. Notice that the mutants' replication is blocked when $h > h_{th,1}$ if $w < w_{th}$, and when $h > h_{th,2}$ if $w > w_{th}$. From (5) and (6) it is easy to determine the rate of change of n and h

$$\begin{aligned}\dot{h} =& \lambda_w h(1 - h)\,[r - 1]\,\theta(R_0 - n) + \\ & + \lambda_w R_0 \frac{h(1 - h)}{n}[r(\theta(h_{th,1} - h)\theta(w_{th} - w) + \\ & + \theta(h_{th,2} - h)\theta(w - w_{th})) - 1]\theta(n - R_0)\end{aligned} \tag{7}$$

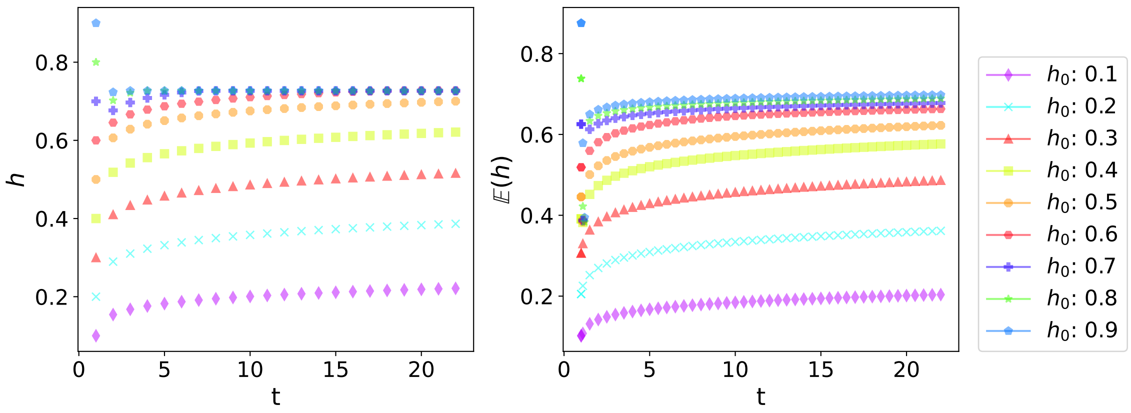

Figure S11: Heteroplasmy (left panel) and mean heteroplasmy (right panel) dynamics of the $H = 5$ model, with parameters corresponding to the best performing parametrization ($\lambda_m = 14, \lambda_w = 12, R_0 = 4.3 \cdot 10^2, h_{th1} = 0.39, h_{th2} = 0.73, w_{th} = 1.0 \cdot 10^2$) and for various choices of h_0 (key).

$$\begin{aligned}\dot{n} =&\lambda_w(rm + w)\theta(R_0 - n)\\ &+R_0\lambda_w\{h[r(\theta(h_{th,1} - h)\theta(w_{th} - w)+\\ &+\theta(h_{th,2} - h)\theta(w - w_{th})) - 1] + 1\}\end{aligned} \tag{8}$$

where $r = \lambda_m/\lambda_w$ is the mutants' relative advantage.

The ODE system eqs. (7), (8) does not admit an analytical solution and was therefore simulated with different choices of the initial conditions and with parameters corresponding to the best parametrization[1] (Fig. S11). It is possible, though, to make some statements about the behavior of h and n after the exponential phase that is only a short transient and therefore does not determine the long–term dynamics. First of all we stress that the behavior of heteroplasmy and copy number depends on the relative value of initial heteroplasmy h_0 and the two thresholds $h_{th,1}, h_{th,2}$. Indeed, h_0 could be (a) larger than the two thresholds, (b) between $h_{th,1}$ and $h_{th,2}$ or (c) smaller than both[2].

(a) $h_0 > h_{th,2} > h_{th,1}$

In this case heteroplasmy decreases and reaches $h_{th,1}$, then is constant

[1] Instead of the Heaviside step function $\theta(h_{th} - h)$ we have used a steep sigmoid function $1/(1 + \exp(-1300(h_{th} - h))$ for numerical stability.

[2] Remember that $h_{th,2} > h_{th,1}$ by design.

until $w = w_{th}$. After that, h increases again to hit $h_{th,2}$. Transformed heteroplasmy h' is then always negative, but starts increasing after $w = w_{th}$.

(b) $h_{th,1} < h_0 < h_{th,2}$
Heteroplasmy behaves in quantitatively similar way to the previous scenario. The main difference is that h' is negative when $w < w_{th}$ and increases to become positive afterwards.

(c) $h_0 < h_{th,1} < h_{th,2}$
In this picture heteroplasmy increases to reach $h_{th,1}$ and then remains constant until $w < w_{th}$. After that, h keeps increasing again until it hits $h_{th,1}$, and again remains constant afterwards.

In all these cases n experiences a linear–like growth. Also, notice that since $n \sim t$, $\dot{h} \leq \frac{\lambda_m R_0}{n} \sim \frac{\lambda_m R_0}{t}$, meaning heteroplasmy increases at a slower rate as the number of molecules grows. To understand why, it is sufficient to remember that the overall number of replicating molecules is fixed at a value R_0, so while the total number of molecules grows over time, the ratio $R_0/n(t)$ decreases and so does the ability of the new molecules to affect the value of the heteroplasmy.

Let us now look at the numerical solutions of eqs. (7) and (8). In Fig. S11 we can see the time evolution of heteroplasmy, simulated in both the deterministic and the stochastic setting (in the latter case mean quantities are represented) with the parameters corresponding to the best parametrization, for different values of the initial heteroplasmy h_0 and with $n_0 = 200$. We can see that there is strong agreement between the two cases.

REFERENCES AND NOTES

1. J. B. Stewart, P. F. Chinnery, The dynamics of mitochondrial DNA heteroplasmy: Implications for human health and disease. *Nat. Rev. Genet.* **16**, 530–542 (2015).

2. D. C. Wallace, Mitochondrial genetic medicine. *Nat. Genet.* **50**, 1642–1649 (2018).

3. G. S. Gorman, A. M. Schaefer, Y. Ng, N. Gomez, E. L. Blakely, C. L. Alston, C. Feeney, R. Horvath, P. Yu-Wai-Man, P. F. Chinnery, R. W. Taylor, D. M. Turnbull, R. McFarland, Prevalence of nuclear and mitochondrial DNA mutations related to adult mitochondrial disease. *Ann. Neurol.* **77**, 753–759 (2015).

4. W. Wei, S. Tuna, M. J. Keogh, K. R. Smith, T. J. Aitman, P. L. Beales, D. L. Bennett, D. P. Gale, M. A. K. Bitner-Glindzicz, G. C. Black, P. Brennan, P. Elliott, F. A. Flinter, R. A. Floto, H. Houlden, M. Irving, A. Koziell, E. R. Maher, H. S. Markus, N. W. Morrell, W. G. Newman, I. Roberts, J. A. Sayer, K. G. C. Smith, J. C. Taylor, H. Watkins, A. R. Webster, A. O. M. Wilkie, C. Williamson; NIHR BioResource–Rare Diseases; 100,000 Genomes Project–Rare Diseases Pilot, S. Ashford, C. J. Penkett, K. E. Stirrups, A. Rendon, W. H. Ouwehand, J. R. Bradley, F. L. Raymond, M. Caulfield, E. Turro, P. F. Chinnery, A. P. Huissoon, A. Crisp-Hihn, A. C. Shaw, A. J. Mead, A. P. Levine, A. J. Thrasher, A. Bierzynska, A. Hassan, A. Kumar, A. Sanchis-Juan, A. Richter, A. Lawrie, A. J. Frary, A. H. Nemeth, A. Olschewski, A. C. Themistocleous, A. C. Browning, A. D. Mumford, A. M. Schaefer, A. Marshall, A. O. M. Wilkie, A. Peacock, A. R. Harper, A. R. Webster, A. S. C. Rice, A. Pyle, A. Koziell, A. M. Drazyk, A. M. Kelly, A. Wagner, A. Attwood, A. de Soyza, A. M. Vandersteen, A. T. Moore, A. Vonk Noordegraaf, A. Rao, A. Herwadkar, A. Houweling, A. Sen, A. Rendon, A. Worth, B. Girerd, B. Madan, B. T. Wilson, C. B. Diz, C. Treacy, C. Brewer, C. Campbell, C. Millar, C. Roughley, C. Titterton, C. Williamson, C. J. Compton, C. Danesino, C. Thys, C. Hadinnapola, C. Deshpande, C. H. Toh, C. van Geet, C. Babbs, C. G. Woods, C. J. Penkett, C. Watt, C. Harris, C. Lentaigne, C. Palles, C. Searle, C. Pilkington, C. Church, C. E. French, C. Samarghitean, D. M. Layton, D. G. Evans, D. Smedley, D. Greene, D. Hart, D. P. Gale, D. G. Kiely, D. Gosal, D. J. Allsup, D. L. Bennett, D. Montani, D. Parry, D. Thomas, D. M. Ruddy, D. Whitehorn, D. Grozeva, D. Bockenhauer, D. Kumararatne, D. Josifova, E. R. Maher, E. K. S. Wong, E. F. Dewhurst, E. Louka, E. Colby, E. Ormondroyd, E. Thomas, E. Swietlik, E. Staples, E. Matthews, E. Woodward, E. Turro, E. Haque, F. L. Raymond, F. Hu, F. Lalloo, F. Soubrier, F.

Cheng, F. A. Flinter, G. Kovacs, G. Arno, G. Hudson, G. Sayer, G. Carr-White, G. Coghlan, G. Evans, G. C. Black, G. Hayman, H. T. Cook, H. Alachkar, H. L. Allen, H. Kazkaz, H. Stark, H. U. Marschall, H. Bogaard, H. Maxwell, H. E. Baxendale, H. L. Hanson, H. Gall, H. Houlden, H. Longhurst, H. Fassihi, H. Olschewski, H. A. Ghofrani, H. S. Markus, H. Watkins, I. P. Tomlinson, I. Simeoni, I. Roberts, J. D. M. Edgar, J. S. R. Gibbs, J. E. Thaventhiran, J. Fox, J. S. Ware, J. Whitworth, J. Collins, J. Suntharalingam, J. Jolley, J. Martin, J. O'Sullivan, J. C. Taylor, J. Chambers, J. Maimaris, J. Clayton-Smith, J. Pepke-Zaba, J. Graham, J. A. Sayer, J. P. Westwood, J. Burn, J. Davis, J. Wharton, J. Taylor, J. Hoffman, J. Stephens, J. Adlard, J. von Ziegenweidt, J. Wessels, K. R. Ong, K. Edwards, K. Downes, K. Gibson, K. Talks, K. Thomson, K. Peerlinck, K. R. Smith, K. Yates, K. E. Stirrups, K. Freson, K. Snape, K. Gomez, K. Sibson, K. W. Muir, K. E. S. Poole, K. G. C. Smith, K. Carss, K. J. Marchbank, K. C. Gilmour, K. Harkness, L. Abulhoul, L. Scelsi, L. Robert, L. E. Lorenzo, L. Izatt, L. Side, L. Wedderburn, L. S. Howard, L. Greenhalgh, M. Chitre, M. A. Kurian, M. Humbert, M. Tischkowitz, M. A. K. Bitner-Glindzicz, M. C. Estiu, M. Erwood, M. Scully, M. Caulfield, M. Gurnell, M. I. McCarthy, M. Toshner, M. Bleda, M. Vazquez-Lopez, M. R. Wilkins, M. Mathias, M. Brown, M. C. Sims, M. Hall, M. J. Daniels, M. S. Buckland, M. Traylor, M. Haimel, M. Cleary, M. Dattani, M. Eyries, M. M. Y. Chan, M. N. Ekani, M. Irving, M. A. Laffan, M. Gattens, M. J. Browning, M. Newnham, M. Simpson, M. Wright, M. Michaelides, M. Lambert, M. Saleem, M. J. Thomas, M. Mozere, M. Ahmed, N. C. Brod, N. Kingston, N. Shah, N. Jurkute, N. Cooper, N. W. Morrell, N. S. Curry, N. Burrows, N. Koelling, N. B. Roy, O. Shamardina, O. Spasic-Boskovic, O. Sadeghi-Alavijeh, P. Gresele, P. F. Chinnery, P. F. K. Yong, P. Yu-Wai-Man, P. A. Lyons, P. Aurora, P. Brennan, P. Corris, P. McAlinden, P. J. Rayner-Matthews, P. Gordins, P. Elliott, P. H. Dixon, P. Kelleher, P. W. Collins, P. Syrris, P. L. Beales, P. Ancliff, P. Griffiths, P. Twiss, P. Yu, Q. Waisfisz, R. A. Floto, R. C. Tait, R. J. Buchan, R. Linger, R. Kazmi, R. B. Sargur, R. Favier, R. Y. Y. Tan, R. Antrobus, R. Quinton, R. Scott, R. Trembath, R. Horvath, R. N. Sarkany, R. Ross-Russell, R. V. MacKenzie Ross, R. Condliffe, R. James, R. Hague, R. Mapeta, R. Armstrong, R. Casey, S. Noorani, S. Tuna, S. A. Johnson, S. Malka, S. Obaji, S. Boyce, S. Goddard, S. J. Rose, S. K. Westbury, S. Mangles, S. Mehta, S. Hackett, S. Moledina, S. Rahman, S. N. Mohammed, S. Banka, S. Holden, S. Pearce, S. Satchell, S. Staines, S. Savic, S. Patel, S. Douzgou, S. Grigoriadou, S. Papadia, S. Ashford, S. Schulman, S. M. Park, S. V. V. Deevi, S. Gräf, S. Abbs, S. J. Wort, S. Jolles, S. Marks, S. Okoli, S. Cook, S. Meacham, S. M. Walker, S. E. Shapiro, S. Sivapalaratnam, T. W. Kuijpers, T. Bariana, T. Bakchoul, T. Everington, T. Renton, T.

Bueser, T. Dent, T. J. Aitman, T. Biss, T. Dutt, T. Fowler, T. Vale, T. Lester, T. R. P. Cole, V. Ganesan, W. A. C. Sewell, W. Wei, W. N. Erber, W. Seeger, W. Kelsall, W. H. Ouwehand, W. Egner, W. G. Newman, W. M. Hague, Y. Wood, Germline selection shapes human mitochondrial DNA diversity. *Science* **364**, (2019).

5. V. I. Floros, A. Pyle, S. Dietmann, W. Wei, W. C. W. Tang, N. Irie, B. Payne, A. Capalbo, L. Noli, J. Coxhead, G. Hudson, M. Crosier, H. Strahl, Y. Khalaf, M. Saitou, D. Ilic, M. A. Surani, P. F. Chinnery, Segregation of mitochondrial DNA heteroplasmy through a developmental genetic bottleneck in human embryos. *Nat. Cell Biol.* **20**, 144–151 (2018).

6. L. M. Cree, D. C. Samuels, S. C. de Sousa Lopes, H. K. Rajasimha, P. Wonnapinij, J. R. Mann, H. H. M. Dahl, P. F. Chinnery, A reduction of mitochondrial DNA molecules during embryogenesis explains the rapid segregation of genotypes. *Nat. Genet.* **40**, 249–254 (2008).

7. T. Wai, D. Teoli, E. A. Shoubridge, The mitochondrial DNA genetic bottleneck results from replication of a subpopulation of genomes. *Nat. Genet.* **40**, 1484–1488 (2008).

8. C. Freyer, L. M. Cree, A. Mourier, J. B. Stewart, C. Koolmeister, D. Milenkovic, T. Wai, V. I. Floros, E. Hagström, E. E. Chatzidaki, R. J. Wiesner, D. C. Samuels, N. G. Larsson, P. F. Chinnery, Variation in germline mtDNA heteroplasmy is determined prenatally but modified during subsequent transmission. *Nat. Genet.* **44**, 1282–1285 (2012).

9. M. S. Sharpley, C. Marciniak, K. Eckel-Mahan, M. McManus, M. Crimi, K. Waymire, C. S. Lin, S. Masubuchi, N. Friend, M. Koike, D. Chalkia, G. MacGregor, P. Sassone-Corsi, D. C. Wallace, Heteroplasmy of mouse mtDNA is genetically unstable and results in altered behavior and cognition. *Cell* **151**, 333–343 (2012).

10. W. Fan, K. G. Waymire, N. Narula, P. Li, C. Rocher, P. E. Coskun, M. A. Vannan, J. Narula, G. R. MacGregor, D. C. Wallace, A mouse model of mitochondrial disease reveals germline selection against severe mtDNA mutations. *Science* **319**, 958–962 (2008).

11. J. B. Stewart, C. Freyer, J. L. Elson, A. Wredenberg, Z. Cansu, A. Trifunovic, N. G. Larsson, Strong purifying selection in transmission of mammalian mitochondrial DNA. *PLoS Biol.* **6**, e10 (2008).

12. J. H. K. Kauppila, H. L. Baines, A. Bratic, M. L. Simard, C. Freyer, A. Mourier, C. Stamp, R. Filograna, N. G. Larsson, L. C. Greaves, J. B. Stewart, A phenotype-driven approach to generate mouse models with pathogenic mtDNA mutations causing mitochondrial disease. *Cell Rep.* **16**, 2980–2990 (2016).

13. M. L. Simard, A. Mourier, L. C. Greaves, R. W. Taylor, J. B. Stewart, A novel histochemistry assay to assess and quantify focal cytochrome c oxidase deficiency. *J. Pathol.* **245**, 311–323 (2018).

14. S. Finnila, S. Tuisku, R. Herva, K. Majamaa, A novel mitochondrial DNA mutation and a mutation in the Notch3 gene in a patient with myopathy and CADASIL. *J. Mol. Med.* **79**, 641–647 (2001).

15. R. McFarland, H. Swalwell, E. L. Blakely, L. He, E. J. Groen, D. M. Turnbull, K. M. Bushby, R. W. Taylor, The m.5650G > A mitochondrial tRNA(A1a) mutation is pathogenic and causes a phenotype of pure myopathy. *Neuromuscul. Disord.* **18**, 63–67 (2008).

16. R. Filograna, C. Koolmeister, M. Upadhyay, A. Pajak, P. Clemente, R. Wibom, M L Simard, A. Wredenberg, C. Freyer, J B Stewart, N G Larsson, Modulation of mtDNA copy number ameliorates the pathological consequences of a heteroplasmic mtDNA mutation in the mouse. *Sci. Adv.* **5**, eaav9824 (2019).

17. K. Hayashi, H. Ohta, K. Kurimoto, S. Aramaki, M. Saitou, Reconstitution of the mouse germ cell specification pathway in culture by pluripotent stem cells. *Cell* **146**, 519–532 (2011).

18. Y. Ohinata, M. Sano, M. Shigeta, K. Yamanaka, M. Saitou, A comprehensive, non-invasive visualization of primordial germ cell development in mice by the Prdm1-mVenus and Dppa3-ECFP double transgenic reporter. *Reproduction* **136**, 503–514 (2008).

19. P. Wonnapinij, P. F. Chinnery, D. C. Samuels, The distribution of mitochondrial DNA heteroplasmy due to random genetic drift. *Am. J. Hum. Genet.* **83**, 582–593 (2008).

20. P. Wonnapinij, P. F. Chinnery, D. C. Samuels, Previous estimates of mitochondrial DNA mutation level variance did not account for sampling error: Comparing the mtDNA genetic bottleneck in mice and humans. *Am. J. Hum. Genet.* **86**, 540–550 (2010).

21. J. P. Burgstaller, I. G. Johnston, N. S. Jones, J. Albrechtová, T. Kolbe, C. Vogl, A. Futschik, C. Mayrhofer, D. Klein, S. Sabitzer, M. Blattner, C. Gülly, J. Poulton, T. Rülicke, J. Piálek, R. Steinborn, G. Brem, MtDNA segregation in heteroplasmic tissues is common in vivo and modulated by haplotype differences and developmental stage. *Cell Rep.* **7**, 2031–2041 (2014).

22. C. W. Birky Jr., Relaxed and stringent genomes—Why cytoplasmic genes dont obey Mendels laws. *J. Hered.* **85**, 355–365 (1994).

23. J. P. Burgstaller, T. Kolbe, V. Havlicek, S. Hembach, J. Poulton, J. Piálek, R. Steinborn, T. Rülicke, G. Brem, N. S. Jones, I. G. Johnston, Large-scale genetic analysis reveals mammalian mtDNA heteroplasmy dynamics and variance increase through lifetimes and generations. *Nat. Commun.* **9**, 2488 (2018).

24. L. Veselovska, S. A. Smallwood, H. Saadeh, K. R. Stewart, F. Krueger, S. Maupetit-Méhouas, P. Arnaud, S. I. Tomizawa, S. Andrews, G. Kelsey, Deep sequencing and de novo assembly of the mouse oocyte transcriptome define the contribution of transcription to the DNA methylation landscape. *Genome Biol.* **16**, 209 (2015).

25. A. Klucnika, H. Ma, A battle for transmission: The cooperative and selfish animal mitochondrial genomes. *Open Biol.* **9**, 180267 (2019).

26. V. Gaysinskaya, A. Bortvin, Flow cytometry of murine spermatocytes. *Curr. Protoc. Cytom.* **72**, 7.44.1–7.44.24 (2015).

27. B. R. Nebel, A. P. Amarose, E. M. Hacket, Calendar of gametogenic development in the prepuberal male mouse. *Science* **134**, 832–833 (1961).

28. I. J. Wilson, P. J. Carling, C. L. Alston, V. I. Floros, A. Pyle, G. Hudson, S. C. E.H. Sallevelt, C. Lamperti, V. Carelli, L. A. Bindoff, D. C. Samuels, P. Wonnapinij, M. Zeviani, R. W. Taylor, H. J. M. Smeets, R. Horvath, P. F. Chinnery, Mitochondrial DNA sequence characteristics modulate the size of the genetic bottleneck. *Hum. Mol. Genet.* **25**, 1031–1041 (2016).

29. Z. Chen, Y. Qi, S. French, G. Zhang, R. C. Garcia, R. Balaban, H. Xu, Genetic mosaic analysis of a deleterious mitochondrial DNA mutation in Drosophila reveals novel aspects of mitochondrial regulation and function. *Mol. Biol. Cell* **26**, 674–684 (2015).

30. B. L. Gitschlag, C. S. Kirby, D. C. Samuels, R. D. Gangula, S. A. Mallal, M. R. Patel, Homeostatic responses regulate selfish mitochondrial genome dynamics in *C. elegans*. *Cell Metab.* **24**, 91–103 (2016).

31. D. K. Howe, D. R. Denver, Muller's ratchet and compensatory mutation in *Caenorhabditis briggsae* mitochondrial genome evolution. *BMC Evol. Biol.* **8**, 62 (2008).

32. H. S. Ma, P. H. O'Farrell, Selfish drive can trump function when animal mitochondrial genomes compete. *Nat. Genet.* **48**, 798–802 (2016).

33. A. B. C. Otten, S. C. E. H. Sallevelt, P. J. Carling, J. C. F. M. Dreesen, M. Drüsedau, S. Spierts, A. D. C. Paulussen, C. E. M. de Die-Smulders, M. Herbert, P. F. Chinnery, D. C. Samuels, P. Lindsey, H. J. M. Smeets, Mutation-specific effects in germline transmission of pathogenic mtDNA variants. *Hum. Reprod.* **33**, 1331–1341 (2018).

34. A. Baracca, G. Sgarbi, M. Mattiazzi, G. Casalena, E. Pagnotta, M. L. Valentino, M. Moggio, G. Lenaz, V. Carelli, G. Solaini, Biochemical phenotypes associated with the mitochondrial ATP6 gene mutations at nt8993. *Biochim. Biophys. Acta* **1767**, 913–919 (2007).

35. J. Van Blerkom, P. W. Davis, J. Lee, ATP content of human oocytes and developmental potential and outcome after in-vitro fertilization and embryo transfer. *Hum. Reprod.* **10**, 415–424 (1995).

36. L. Boulet, G. Karpati, E. A. Shoubridge, Distribution and threshold expression of the tRNA(Lys) mutation in skeletal muscle of patients with myoclonic epilepsy and ragged-red fibers (MERRF). *Am. J. Hum. Genet.* **51**, 1187–1200 (1992).

37. P. May-Panloup, L. Boucret, J. M. Chao de la Barca, V. Desquiret-Dumas, V. Ferré-L'Hotellier, C. Morinière, P. Descamps, V. Procaccio, P. Reynier, Ovarian ageing: The role of mitochondria in oocytes and follicles. *Hum. Reprod. Update* **22**, 725–743 (2016).

38. R. H. Hamalainen, T. Manninen, H. Koivumaki, M. Kislin, T. Otonkoski, A. Suomalainen, Tissue- and cell-type-specific manifestations of heteroplasmic mtDNA 3243A>G mutation in human induced pluripotent stem cell-derived disease model. *Proc. Natl. Acad. Sci. U.S.A.* **110**, E3622–3630 (2013).

39. T. Lieber, S. P. Jeedigunta, J. M. Palozzi, R. Lehmann, T. R. Hurd, Mitochondrial fragmentation drives selective removal of deleterious mtDNA in the germline. *Nature* **570**, 380–384 (2019).

40. S. P. Burr, M. Pezet, P. F. Chinnery, Mitochondrial DNA heteroplasmy and purifying selection in the mammalian female germ line. *Develop. Growth Differ.* **60**, 21–32 (2018).

41. A. Pyle, R. W. Taylor, S. E. Durham, M. Deschauer, A. M. Schaefer, D. C. Samuels, P. F. Chinnery, Depletion of mitochondrial DNA in leucocytes harbouring the 3243A->G mtDNA mutation. *J. Med. Genet.* **44**, 69–74 (2007).

42. J. Griffin, B. R. Emery, I. Huang, C. M. Peterson, D. T. Carrell, Comparative analysis of follicle morphology and oocyte diameter in four mammalian species (mouse, hamster, pig, and human). *J. Exp. Clin. Assist. Reprod.* **3**, 2 (2006).

43. J. Spiropoulos, D. M. Turnbull, P. F. Chinnery, Can mitochondrial DNA mutations cause sperm dysfunction? *Mol. Hum. Reprod.* **8**, 719–721 (2002).

44. C. Calabrese, D. Simone, M. A. Diroma, M. Santorsola, C. Guttà, G. Gasparre, E. Picardi, G. Pesole, M. Attimonelli, MToolBox: A highly automated pipeline for heteroplasmy annotation and prioritization analysis of human mitochondrial variants in high-throughput sequencing. *Bioinformatics* **30**, 3115–3117 (2014).

45. C. Gu, S. Liu, Q. Wu, L. Zhang, F. Guo, Integrative single-cell analysis of transcriptome, DNA methylome and chromatin accessibility in mouse oocytes. *Cell Res.* **29**, 110–123 (2019).

46. F. A. Wolf, P. Angerer, F. J. Theis, SCANPY: Large-scale single-cell gene expression data analysis. *Genome Biol.* **19**, 15 (2018).

47. H. Mi, D. Ebert, A. Muruganujan, C. Mills, L.-P. Albou, T. Mushayamaha, P. D. Thomas, PANTHER version 16: A revised family classification, tree-based classification tool, enhancer regions and extensive API. *Nucleic Acids Res.* **49**, D394–D403 (2020).

48. T. Konopka, umap: Uniform Manifold Approximation and Projection. R package version 0.2.7.0 (2020); https://CRAN.R-project.org/package=umap.

49. P. F. Chinnery, D. C. Samuels. Relaxed replication of mtDNA: A model with implications for the expression of disease. *Am. J. Hum. Genet.* **64**, 1158–1165 (1999).

50. D. T. Gillespie. A general method for numerically simulating the stochastic time evolution of coupled chemical reactions. *J. Com. Phys.* **22**, 403–434 (1976).

51. M. Jiang, T. E. S. Kauppila, E. Motori, X. Li, I. Atanassov, K. Folz-Donahue, N. A. Bonekamp, S. Albarran-Gutierrez, J. B. Stewart, N. G. Larsson, Increased total mtDNA copy number cures male infertility despite unaltered mtDNA mutation load. *Cell Metab.* **26**, 429–436.e4 (2017).
